# Iodine enriched kale (*Brassica oleracea* var. *sabellica* L.)—The influence of heat treatments on its iodine content, basic composition and antioxidative properties

**DOI:** 10.1371/journal.pone.0304005

**Published:** 2024-06-27

**Authors:** Joanna Krzemińska, Joanna Kapusta-Duch, Sylwester Smoleń, Iwona Kowalska, Jacek Słupski, Radosława Skoczeń-Słupska, Katarzyna Krawczyk, Justyna Waśniowska, Aneta Koronowicz

**Affiliations:** 1 Department of Human Nutrition and Dietetics, Faculty of Food Technology, University of Agriculture in Krakow, Krakow, Poland; 2 Department of Plant Biology and Biotechnology, Faculty of Biotechnology and Horticulture, University of Agriculture in Krakow, Krakow, Poland; 3 Department of Plant Product Technology and Nutrition Hygiene, Faculty of Food Technology, University of Agriculture in Krakow, Krakow, Poland; Institute for Biological Research, University of Belgrade, SERBIA

## Abstract

Iodine deficiency in the diet globally continues to be a cause of many diseases and disabilities. Kale is a vegetable that has health-promoting potential because of many nutrients and bioactive compounds (ascorbic acid, carotenoids, glucosinolates and phenolic compounds). *Brassica* vegetables, including kale, have been strongly recommended as dietary adjuvants for improving health. The nutrient and health-promoting compounds in kale are significantly affected by thermal treatments. Changes in phytochemicals upon such activities may result from two contrary phenomena: breakdown of nutrients and bioactive compounds and a matrix softening effect, which increases the extractability of phytochemicals, which may be especially significant in the case of iodine-fortified kale. This study investigated changes of basic composition, iodine, vitamin C, total carotenoids and polyphenols contents as well as antioxidant activity caused by steaming, blanching and boiling processes in the levels of two cultivars of kale (green and red) non-biofortified and biofortified via the application to nutrient solutions in hydroponic of two iodoquinolines [8-hydroxy-7-iodo-5-quinolinesulfonic acid (8-OH-7-I-5QSA) and 5-chloro-7-iodo-8-quinoline (5-Cl-7-I-8-Q)] and KIO_3_. Thermal processes generally significantly reduced the content of the components in question and the antioxidant activity of kale, regardless of cultivar and enrichment. It was observed that the red cultivar of kale had a greater ability to accumulate and reduce iodine losses during the culinary processes. 8-hydroxy-7-iodo-5-quinolinesulfonic acid showed a protective effect against the treatments used, compared to other enrichments, thus contributing to the preservation of high iodine content.

## Introduction

Noncommunicable diseases (NCDs) are usually long lasting and are a result of a combination of genetic, physiological, environmental and behavioural causes. NCDs kill 41 million people each year, equivalent to 74% of all deaths globally [1–3]. Diet has a key role in the prevention and management of chronic non-communicable diseases. Functional food, or the so-called FOSHU (Foods for Specified Health Use) is normal food from which harmful ingredients (e.g. allergens) have been removed or which has been enriched with physiologically active substances to produce a product with sufficient nutritional value to improve human health [4].

Numerous studies point to the importance of vegetables and fruits as the richest source of bioactive compounds in the daily human diet and indicate that a diet rich in plant-based products reduces the risk of developing chronic non-communicable diseases [5, 6]. Biofortification of vegetables can improve the nutritional status of different population groups, prevent malnutrition due to iodine deficiency, and reduce the risk of NCDs [1]. Iodine is a very important element for human health. The thyroid gland needs iodine for the synthesis of thyroid hormones. Iodine deficiency results in insufficient thyroxine production and associated thyroid, metabolic, developmental and reproductive disorders [7]. Iodine supplementation is usually necessary to prevent iodine deficiency disorders (IDD), especially in endemic areas [1]. Iodised salt is the cornerstone of iodine prophylaxis in endemic areas and continuous monitoring of community iodine intake and associated clinical outcomes is essential [7, 8]. On the other hand, findings by Hao et al. [9] indicate that a lower frequency of salt added to food is associated with a lower risk of cardiovascular disease.

Kale (*Brassica oleracea* var. *sabellica* L.) is a very healthy vegetable and the additional biofortification with iodine makes its chemical composition even more attractive. Green and red kale leaves are an excellent source of bioactive compounds: carotenoids, especially β-carotene and lutein, dietary fibre (pectin), glucosinolates, as well as polyphenolic compounds. In addition, kale contains high amounts of vitamins such as C, A, K and folic acid [10–13]. Moreover, sulforaphane (SFN), indole-3-carbinol (I3C) and iberin may help to reduce the risk of oestrogen-sensitive cancer as well as other types of cancer, such as prostate, liver, colorectal, melanoma, and pancreas [14–20]. Its consumption has increased in recent years but it is still not high enough. Usually, kale is considered a vegetable for garnishing dishes and not a food with high values for human health. In recent years, research interest in kale has increased in the context of its anti-cancer properties. Increased intake of this vegetable, in the form of salads, smoothies, or cocktails, is particularly recommended for oncology patients, those with cardiovascular and skeletal disorders and many others. It is also worth promoting the addition of kale to soups, making kale chips (in the oven), using it as a burger topping, interchangeable with lettuce, or as a crust for casseroles or burritos [12, 13].

Iodine is a non-essential element for higher plants, including kale leaves. However, all plants can absorb it from the soil. The ability of the soil to absorb iodine influences the decision to fertilise with it. In clay and humus soils, iodine is bound by iron, aluminium oxides, and organic matter. Therefore, plants grown on these soils have a low iodine content [21]. Iodine fertilisation of this soil type is less efficient in terms of its uptake, compared to sandy soils and water crops [21–24].

Iodine is better stored in vegetative organs such as roots, stems, and leaves [24, 25], suggesting differences in iodine accumulation between organs in plants, including kale [26, 27]. Plants bind iodine in proteins used in many important biological processes. These include the Rubisco protein, which allows efficient photosynthesis in leaves, peroxidases, which protect plants from abiotic and biotic stresses, and ATPases, which support the energy supply needed for growth and nutrient transport. It is anticipated that iodine deficiency in plants can cause yield losses similar to those resulting from deficiencies of any other nutrients. However, at higher concentrations iodine in plants, including leaves of kale, can be toxic, leading to leaf damage (chlorosis and necrosis) and stunted plant growth [28–30].

There is an ever-increasing interest among researchers to conduct studies on the effects of biofortification with iodine and heat treatments on the content of iodine, basic composition, bioactive compounds as well as antioxidants of kale. This is confirmed by studies by Krawczyk et al. (2024) [31] on the effect of biofortification of kale with iodoquinolines and by Waśniowska et al. (2023) [32] examining the effects of using heat treatment on iodine-biofortified kale.

To summarise, the final concentration of bioactive compounds before and after heat treatments was analysed many times to assess their availability in the diet. But novelty of this study lies in the fact that research on incorporation of iodine (in the form of potassium iodate and iodoquinolines) directly into plants (especially vegetables, including kale) through hydroponic cultivation has received little attention until now. In this study, the effect of steaming, blanching and boiling without salt on iodine concentrations in kale biofortified with this element was assessed for the first time. A lot of popular the vegetables, including kale, were tested during heat treatment; however, how heat treatment affects the levels of the newly introduced compounds has not been tested and needs to be investigated before consumption by consumers. So far never been tested kale biofortified with KIO_3_, 8-hydroxy-7-iodo-5-quinolinesulfonic acid and 5-chloro-7-iodo-8-quinolinol. This study analysed changes in basic composition and iodine, dietary fibre, total carotenoids and total polyphenols content as well as the antioxidant activity of kale after different heating treatments.

## Materials and methods

### Plant material and growth conditions

Hydroponic cultivation of curly kale (*Brassica oleracea* var. *sabellica* L.) two cultivars ‘Oldenbor F_1_’ (green kale) and ‘Redbor F_1_’ (red kale) was conducted in the spring of 2022. The experiments were located in the greenhouse at the campus of the Faculty of Biotechnology and Horticulture of the University of Agriculture in Kraków (50◦0500300 N 19◦5700100 E). The seeds were sown into 96-cell propagation trays with 42x42x65 mm sized cells filled with vermiculite. Seedlings in phase 3 true leaves were transplanted into the hydroponic NFT (Nutrient Film Technique) system. The seedlings were placed in trays with holes at 30 cm intervals. The plants were grown in the so-called ’dry hydroponic’ NFT (without substrate). The plants were watered at 5:00–19:00 and 1:00–2:00, 1 minute in 5-minute intervals, during day and night, respectively. The nutrient solution used to grow the curly kale contained micro- and macro-nutrients at concentrations (mg/L) of N-130, P-50, K-220, Ca-120, Mg-45, Fe-2, Mn-0.55, B-0.33, Zn-0.33, Cu-0.15, M- 0.05 and pH 5.70 adjusted with 38% nitric acid. A final EC (Electric Conductivity) of 2.0 mS-cm^-1^ was obtained.

### Biofortification treatment

The following treatments were included in the experiment: Control and KIO_3_, 8-hydroxy-7-iodo-5-quinolinesulfonic acid (8-OH-7-I-5QSA) and 5-chloro-7-iodo-8-quinolinol (5-Cl-7-I-8-Q). Their application at 10 μM concentration (introduced into the solution with other ingredients, as described above) started at the stage of 4–5 true leaves and was continuous until the harvest of the plants. There were 4 replicates, 6 plants per each replicate and 24 plants per one combination of each cultivars, in total 120 plants of each cultivar in the experiment. The plants were harvested on the 63rd day of cultivation when the kale had 13–14 true leaves.

### Harvesting, preliminary processing, and culinary treatment of kale

In spring 2022, the kale was harvested in the greenhouse, segregated by experimental groups, weighed and transported to the laboratory for further analyses. The leaves were separated from the stems. This was done to extract the parts edible by consumers. The leaves were subjected to heat treatment, i.e., steaming, blanching, and boiling.

The steaming process was carried out in a combi steamer (Retigo Orange Version 6 × GN1/1|O 611, Rožnov, Czech Republic). Curly kale leaves were placed on stainless steel metal trays, with one layer of this vegetable on each tray. They were steamed at 100°C for about 15 minutes. They were evaporated until they were tender for consumption, that is, until the kale became soft and easy to chew (15 minutes).

Blanching was carried out in distilled water using an electric cooker (Mastercook KE 2003 B Dynamic, Wrocław, Poland). Distilled water was used to correctly determine the iodine content without false positives. A portion (500 g of leaves for 1 L of distilled water) of curly kale leaves was placed in a stainless steel metal pot. The kale was immersed in water at 85–95°C and slightly boiled for about 3–5 min. The boiling water was poured out and then the kale was rinsed with cold distilled water.

The kale was cooked in distilled water using an electric cooker (Mastercook KE 2003 B Dynamic, Wrocław, Poland). The distilled water was used to correctly determine the iodine content without false positives. The kale was boiled in water in a stainless steel metal pot. The water was brought to the boil and the kale (500 g) was cooked for 20 minutes in 1 L of water until tender for consumption. The boiling process was carried out without a lid to evaporate the glucosinolates.

During the boiling processes, the weight ratio of the kale before and after the boiling process was taken into account. The following formula was used to determine the content of the individual ingredients, taking into account the mass balance.

$$\text{TR}\left[\%\right]=\left(\text{Nc}\;\text{Gc}\right)/\left(\text{Nr}\;\text{Gr}\right)100$$

Nc = nutrient content per gram of heat-treated food;

Gc = gram of heat-treated food;

Nr = nutrient content per gram of raw food;

Gr = gram of food before heat treatment.

Water after boiling was taken for iodine analyses. During kale boiling, the pre- (1L) and post- (about 400 mL) boiling water balances were determined in order to determine how much water evaporated during the process. On average, about 60% of the water evaporated during the boiling process.

### Preparation of kale samples

A standardized sample of fresh kale leaves (separately for treatments and replications) was frozen at -20°C and then subsequently lyophilised using a Christ Alpha Gamma 1–16 LSCplus lyophiliser (Martin Christ Gefriertrocknungsanlagen GmbH, Harz, Germany) and ground in a laboratory hurricane mill (WZ-1, Sadkiewicz Instruments, ZDPP, Poland). Samples were stored in sealed polyethylene bags (at 2–8°C) until the analysis. The samples were subsequently analysed to determine the concentrations of iodine, by using the ICP-MS/MS technique (Inductively coupled plasma–triple quadrupole mass spectrometry; iCAP TQ ICP-MS; ThermoFisher Scientific, Bremen, Germany). In the dry material, iodine, total nitrogen (N-total), total fats, total fibre and ash were determined. Methodologies are described below. The results were calculated and presented relative to fresh weight of the kale.

### Determination of total iodine

The iodine content in freeze-dried kale leaves samples (fresh and after culinary processes) was analysed via the alkaline extraction of 0.2 g of the samples with tetramethylammonium hydroxide (TMAH), with the use of amylase to digest starch (during the extraction process), and by using inductively coupled plasma mass spectrometry (ICP-MS/MS) with a triple quadruple spectrometer (iCAP TQ ICP-MS). The analysis was based on research published by Smoleń et al. [33], based on PN-EN 15111:2008 [34]. In total, 0.2 g of air-dried leaves kale samples, 10 mL of double distilled water and 1 mL of 25% TMAH (Sigma-Aldrich) were put into 30 mL Falcon tubes. After adequate mixing, the samples were incubated for 3 hours at 90°C. Samples were cooled after incubation to approximately 20°C and made up to 30 mL with double distilled water. After mixing, the samples were centrifuged for 15 min at 4500 rpm. Iodine content was analysed in the supernatants.

### Analysis of basic chemical composition

The dry matter content in kale was determined using the drying method at 105°C, AOAC 930.04 (Association of Official Analytical Chemists) [35].

The total protein content was measured using the Kjeldahl method (PN-EN ISO 8968–1: 2014–03). The sample of kale was previously lyophilized and then mineralized for about 1 h in concentrated sulphuric acid (VI). Then, the ammonia formed from the nitrogenous bonds contained in the protein was distilled off and titrated with 0.1 M hydrochloric acid in the presence of a Tashiro indicator to a light purple colour (AOAC procedure No. 950.36) [35].

The crude fat content of the kale samples was determined using the Soxhlet method (PN-A-79011-4: 1998) [35]. The fat was extracted using 80 mL of petroleum ether (v/v 40:60). During the analyses, the sample was subjected to repeated continuous extraction using a Foss Soxtec 2050 Solvent Extraction System apparatus (AOAC Procedure No. 950.38).

The mineral content in the form of ash was determined using the method PN-A79011-8: 1998 [35]. Samples were weighed, ashed over a burner, and finally ashed in a muffle furnace (SNOL 8.2/1100, Lithuania). The time and rate of the process were adjusted accordingly so that the organic compounds were ashed and only the minerals forming the crude ash remained (AOAC procedure No. 930.05).

The determination of the total dietary fibre was based on the AACC 32–05.01 method and the AOAC 985.29 method [36]. A sample of the kale was subjected to enzyme digestion (including thermostable α-amylase, purified protease and purified amyloglucosidase). The sample of kale (amount of 0.5 g) was weighed. To each beaker 50 mL phosphate buffer (pH 6.0) and 50 μL heat-stable α-amylase solution were added. The beakers with the sample were boiled in a water bath (98–100°C for 15 min). The samples were adjusted to pH 7.5±0.1 by adding 10 mL 0.275 N NaOH solution and added 100 μL of protease solution. The beakers were incubated at 60°C for 30 min. Then, cooled and 10 mL of 0.325 N HCl solution (to pH to 4.5±0.2) were added to them. After that, 200 μL amyloglucosidase was added and the samples were incubated for 30 min at 60°C. 280 mL 95% EtOH were pre-heated to 60°C and then added. Suction was applied to draw Celite onto the fritted glass as an even mat. The crucibles were residue overnight in 105°C air oven. Samples were weighed to the nearest 0.1 mg. Crucible and Celite weights were subtracted to determine the weight of the residue. One sample of a set of duplicates was analysed for the residue from protein by AOAC procedure No. 950.36 [35], using N x 6.25 as a conversion factor. The second residue sample was incinerated for 5 h at 525°C. Finally, the sample was weighed and crucible and Celite weights were subtracted to determine ash AOAC procedure No. 930.05 [35].

The total carbohydrates are expressed using the following formula [37]:

$$\begin{array}{c} \text{The}\;\text{total}\;\text{carbohydrates}=100–[\text{total}\;\text{fat}\;\left(\text{g}\right)+\text{total}\;\text{protein}\;\left(\text{g}\right)\\ +\text{minerals}\;\text{in}\;\text{the}\;\text{form}\;\text{of}\;\text{ash}\;\left(\text{g}\right)+\text{dietary}\;\text{fibre}\;\left(\text{g}\right)] \end{array}$$

## Determination of the antioxidant capacities

### Preparation of ethanol extracts

The ethanol extracts (10 g of raw curly kale leaves in 40 mL of 96% ethanol solution) were incubated at 95°C for 15 min. They were then cooled, homogenised, and filtered on a medium-quality filter. The filtrate was transferred to a 50 ml flask and made up to volume with 96% ethanol solution, stored at −22°C. The polyphenol content and ABTS^●+^ radical quenching activity were determined in the extract thus prepared.

### Determination of the total phenolic content

The ethanol extracts were used for the determination of total polyphenolic compounds using the Folin-Ciocalteu reagent (Merck, St. Louis, MO, USA). The extracts were diluted 1:20 with distilled water. Phenolic content was measured after 20 min by spectrophotometry at 760 nm (blank = ethanol), using 5 mL diluted extracts, 0.5 mL of Folin-Ciocalteu reagent, and 0.25 mL of 25% sodium carbonate and using a RayLeigh UV-1800 spectrophotometer (China). Results were expressed as milligrams of gallic acid equivalents per 100 g (mg GAE 100 g ^-1^ F.W.) of fresh weight, based on the standard curve for the aforementioned acid [38].

### Determination of the antioxidant activity of ABTS^●+^

Antioxidant potential was determined in ethanol extracts from kale leaves using the ABTS^●+^ radical quenching capacity method. The free radical quenching capacity was expressed as Trolox equivalent in the mg/g of sample. Trolox was purchased from Merck (Merck, St. Louis, MO, USA). The colour change, monitored by the change in absorbance at 734 nm via a spectrophotometer (UV-1800, Rayleigh, Beijing, China) after a specified time and temperature (6 min at 30°C), is proportional to the antioxidant’s concentration [39].

### Determination of the total carotenoids content

The total carotenoid content was analysed by extracting carotenoids from samples using a mixture of acetone and hexane according to the Polish Standard, with minor modifications [40, 41]. Samples from 1 g of fresh kale leaves were extracted in a porcelain mortar with a mixture of acetone and hexane (4:6 v/v), with the addition of approximately 0.5 g of sand roasted in concentrated HCl. The extracts were transferred to a cylinder, and the volume of the extract was measured and then made up to 40 mL in volume. After 30 minutes of storege in the dark, the extracts were diluted 1:10, and absorbance values were measured at 450 nm using a spectrophotometer (UV-1800, Rayleigh, Beijing, China). The resulting values were assessed using a β-carotene standard curve (Merck, St. Louis, MO, USA).

### Determination of L-ascorbic acid

The vitamin C content (ascorbic acid) was determined by capillary electrophoresis in fresh kale leaves. The method was described in an earlier publication by Smoleń et al. (2016) [41]. Two grams of homogenised kale leaves were poured into 8 mL of 2% oxalic acid and then centrifuged for 15 minutes at 4 500 rpm. The supernatant was collected and centrifuged again for 10 min at 10 000 rpm. The aliquot supernatant was analysed using Beckman PA 800 Plus capillary electrophoresis (CE) with DAD detection. Capillaries with an outer diameter of 365 μm, inner diameter of 50 μm, and total length of 50 cm (40 cm to the detector) were used. A negative power supply of -25 kV was used. Buffer broth containing 15 mM Na2B4O7, 30 mM NaH_2_PO_4_, and 0.2 mM CTAB (pH 8.80) was used.

### Assessment of iodine in biofortified kale for consumer health and safety

Percentage of recommended daily intake of iodine (RDA-I) and daily intake of iodine (D-I) were calculated using 50 g and 100 g of kale leaves, respectively. The value of RDA-I was based on the World Health Organization’s (WHO) recommendation for children above 12 years old and adults, i.e., 150 μg of I [42–44]. The average daily dose (ADD) was determined using the following equation:

$$\text{ADD}=\left(\text{MI}\cdot \text{CF}\cdot \text{DI}\right)/\text{BW}$$

MI—the iodine concentration in the leaves of curly kale (mg·kg^−1^ d.w.),

CF—the fresh to dry weight conversion factor for the plant samples (the ratio averages 0.162),

DI—the daily iodine intake (0.1 kg),

BW—the body weight measurement (70 kg).

The consumer safety of iodine-enriched leaves of kale was evaluated on the basis of the hazard quotient (HQ) values that describe the risk to human health that results from the intake of I through the consumption of fresh kale leaves. The values obtained grom the HQ calculation represent only the intake of I from fresh kale leaves. The method of calculation is presented in the publication by Kessler [44]. The HQ-I value was calculated from the equation:

HQ=ADD/RfD

RfD—represents the dietary recommended tolerable upper intake level of I

The RfD value calculated for iodine I was 1100 μg I-day-1 [42, 44].

ADD—the average daily intake of I (mg I per kg body weight per day) [43].

### Statistical analysis

The experimental data were analysed using a three-factor analysis of variance (ANOVA), except content of iodine in water after boiling kale (two-factor). Statistics were performed using the program Statistica 13.0 PL (https://www.tibco.com/products/data-science, accessed 30 July 2023, StatSoft Inc., Tulsa, OK 74104, USA). The level of significance considered was p < 0.05. In the case of significant effects, homogeneous groups were distinguished by Duncan’s post hoc test. All chemical analyses were performed with a minimum of three or four replicates. In the three-factor experiment, the variables were: factor No. 1: culinary treatment (raw, steaming, blanching, boiling), factor No. 2: type of enrichment (KIO_3_, 8-Hydroxy-7-iodo-5-quinolinesulfonic, 5-Chloro-7-iodo-8-quinolinol), factor No. 3: cultivar of kale (‘Oldenbor F_1_’,’Redbor F_1_’), with all possible interactions.

## Results

### Analysis of total iodine in kale

Based on the statistical analyses (Table 1, S1A Fig), it was shown that the process of hydroponic biofortification of kale leaves from two cultivars ‘Oldenbor F_1_‘ and ‘Redbor F_1_’ with inorganic (KIO_3_) and organic (8-OH-7-I-5QSA and 5-Cl-7-I-8-Q) iodine was significant, compared to the control. Raw control kale accumulated iodine at 178.49 μg∙kg^-1^ F.W. (‘Oldenbor F_1_’) and 204.49 μg∙kg^-1^ F.W. (‘Redbor F_1_’). Application of KIO_3_ (as an iodine positive control) affected iodine accumulation at 2442.59 μg∙kg^-1^ F.W. (‘Oldenbor F_1_’) and 2130.97 μg∙kg^-1^ F.W. (‘Redbor F_1_’). Application of iodoquinoline in the form of 8-OH-7-I-5QSA showed statistically significant higher iodine levels of 2097.98 μg∙kg^-1^ F.W. (‘Oldenbor F_1_’) and 2434.70 μg∙kg^-1^ F.W. (‘Redbor F_1_’) compared to the control. Also, the application of 5-Cl-7-I-8-Q statistically significantly affected higher iodine accumulation relative to the control in both kale cultivars 1206.00 μg∙kg^-1^ F.W. (‘Oldenbor F_1_’) and 1034.00 μg∙kg^-1^ F.W. (‘Redbor F_1_’).

**Table 1:** Content of Iodine μg∙kg^-1^ F.W. of curly kale leaves ‘Oldenbor F_1_’ and ‘Redbor F_1_‘ with or without biofortification before and after heat treatment.

Culinary treatment	Enrichment	Cultivar	Iodine μg∙kg^-1^ F.W.
Raw	Control	‘Oldenbor F_1_’	178.49 ± 23.68 ^d^
		‘Redbor F_1_’	204.49 ± 10.84 ^d^
	KIO_3_	‘Oldenbor F_1_’	2442.59 ± 7.22 ^t^
		‘Redbor F_1_’	2130.97 ± 20.73 ^r^
	8-OH-7-I-5QSA	‘Oldenbor F_1_’	2097.98 ± 37.64 ^r^
		‘Redbor F_1_’	2434.70 ± 82.14 ^t^
	5-Cl-7-I-8-Q	‘Oldenbor F_1_’	1206.00 ± 57.45 ^k^
		‘Redbor F_1_’	1034.00 ± 18.18 ^j^
Steaming	Control	‘Oldenbor F_1_’	103.24 ± 3.35 ^c^
		‘Redbor F_1_’	89.11 ± 1.72 ^c^
	KIO_3_	‘Oldenbor F_1_’	1903.96 ± 82.48 ^o^
		‘Redbor F_1_’	1793.85 ± 18.19 ^n^
	8-OH-7-I-5QSA	‘Oldenbor F_1_’	2031.58 ± 29.61 ^p^
		‘Redbor F_1_’	2388.01 ± 20.90 ^s^
	5-Cl-7-I-8-Q	‘Oldenbor F_1_’	987.34 ± 6.59 ^j^
		‘Redbor F_1_’	998.36 ± 31.57 ^j^
Blanching	Control	‘Oldenbor F_1_’	43.41 ± 1.05 ^a^
		‘Redbor F_1_’	33.38 ± 1.31 ^a^
	KIO_3_	‘Oldenbor F_1_’	779.32 ± 35.89 ^h^
		‘Redbor F_1_’	1242.94 ± 15.59 ^kl^
	8-OH-7-I-5QSA	‘Oldenbor F_1_’	1377.32 ± 26.32 ^m^
		‘Redbor F_1_’	1397.83 ± 45.34 ^m^
	5-Cl-7-I-8-Q	‘Oldenbor F_1_’	562.23 ± 13.20 ^f^
		‘Redbor F_1_’	331.62 ± 8.16 ^e^
Boiling	Control	‘Oldenbor F_1_’	51.90 ± 0.78 ^b^
		‘Redbor F_1_’	30.81 ± 1.07 ^a^
	KIO_3_	‘Oldenbor F_1_’	842.13 ± 8.56 ^i^
		‘Redbor F_1_’	658.75 ± 16.05 ^g^
	8-OH-7-I-5QSA	‘Oldenbor F_1_’	745.99 ± 3.61 ^h^
		‘Redbor F_1_’	1287.78 ± 70.53 ^l^
	5-Cl-7-I-8-Q	‘Oldenbor F_1_’	593.71 ± 30.96 ^f^
		‘Redbor F_1_’	299.29 ± 4.24 ^e^

Results are shown as mean ± standard error (SE); n = 4; homogeneous groups refer to a three-factor analysis of variance: factor No. 1 culinary treatment: raw, steaming, blanching, boiling x factor No. 2 type of enrichment: control, KIO_3_, 8-OH-7-I-5QSA, 5-Cl-7-I-8-Qx factor No. 3 kale cultivar: ’Oldenbor F_1_’ and ’Redbor F_1_’; means followed by the same letter are not significantly different *p* < 0.05 (Duncan’s post-hoc test).

### The influence of thermal processes on iodine concentration in kale

Table 1 presents the results of iodine that remained in the leaves of kale of both cultivars after thermal processes (steaming, blanching, and boiling). It was shown that the boiling processes used had a statistically significant effect on reducing the iodine content of kale in both ‘Oldenbor F_1_’ and ‘Redbor F_1_’ cultivars. Statistically significant differences were shown in the iodine content of both kale cultivars after applying the same process. The reduction of iodine levels in kale of both ‘Oldenbor F_1_’ and ‘Redbor F_1_’ cultivars was least affected by the steaming process, next by blanching, and the most by the boiling process.

### Content of iodine in water after boiling kale

Table 2 (S1B Fig) presents the results of iodine content in water after boiling kale. Boiling kale leaves can contribute to the efflux of iodine or its oxidation, which result in the lower concentration in the leaf. The microelement passes into the water, and there it can be detected what portion has entered the broth. There was a statistically significant iodine content in water after boiling, after biofortification than in not biofortified kale leaves of both cultivars. After kale leaves were biofortified with KIO_3_ and 8-OH-7-I-5QSA, there was statistically significantly more iodine in the water after boiling of the ’Redbor F_1_’ cultivar than in the water after boiling of the ’Oldenbor F_1_’ cultivar. The higher the amount of iodine in the leaves, the greater the flow of microparticles into the water, thus the concentration is high in the tested water samples after boiling.

**Table 2:** Iodine content (mg∙L^-1^) of the water after boiling the leaves of curly kale of both cultivars ‘Oldenbor F_1_’ and ‘Redbor F_1_’ with or without biofortification.

Enrichment	Cultivar	Iodine mg∙L^-1^
Control	‘Oldenbor F_1_’	2.48±0.07 ^a^
	‘Redbor F_1_’	3.37±0.04 ^a^
KIO_3_	‘Oldenbor F_1_’	97.84±0.9 ^e^
	‘Redbor F_1_’	112.63±1.24 ^f^
8-OH-7-I-5QSA	‘Oldenbor F_1_’	82.33±0.5 ^c^
	‘Redbor F_1_’	86.09±0.14 ^d^
5-Cl-7-I-8-Q	‘Oldenbor F_1_’	39.06±0.04 ^b^
	‘Redbor F_1_’	38.83±0.4 ^b^

Results are shown as mean ± standard error (SE); n = 2; homogeneous groups refer to two-factor analysis of variance: factor No. 1 type of enrichment: control, KIO_3_, 8-OH-7-I-5QSA, 5-Cl-7-I-8-Q, x factor No. 2 kale cultivar: ’Oldenbor F_1_’ and ’Redbor F_1_’; means followed by the same letter are not significantly different *p* < 0.05 (Duncan’s post-hoc test).

## The effect of thermal processes on dry matter content and basic chemical composition of kale

### Dry matter

Table 3 (S2A Fig) shows the results of dry matter content in the leaves of raw and heat-treated kale. The application of thermal processes significantly affected the dry matter content of the biofortified kale cultivars tested, compared to the control. This is likely due to the steaming of water from the kale leaf structure during the steaming process and, consequently, the concentration of ingredients, which increased dry matter content.

**Table 3:** Chemical composition of kale ‘Oldenbor F_1_’ and ‘Redbor F_1_’ in fresh tissue before and after heat treatment.

Culinary treatment	Enrichment	Cultivar	Dry matter g∙100 g^-1^ F.W.	Ash g∙100 g^-1^ F.W.	Protein g∙100 g^-1^ F.W.	Fat g∙100 g^-1^ F.W.	Total Carbohydrate g∙100 g^-1^ F.W.	Dietary fibre g∙100 g^-1^ F.W.
Raw	Control	‘Oldenbor F_1_’	10.30 ± 0.22 ^h^	2.07 ± 0.02 ^rs^	3.39 ± 0.01 ^h^	0.56 ± 0.02 ^efg^	4.78 ± 0.04 ^i^	3.00 ± 0.08 ^efgh^
		‘Redbor F_1_’	10.10 ± 0.23 ^h^	2.11 ± 0.01 ^t^	2.58 ± 0.23 ^g^	0.32 ± 0.01 ^c^	5.13 ± 0.22 ^jk^	3.60 ± 0.04 ^ijk^
	KIO_3_	‘Oldenbor F_1_’	10.50 ± 0.24 ^h^	2.06 ± 0.01 ^rs^	3.24 ± 0.11 ^h^	0.52 ± 0.02 ^e^	4.99 ± 0.13 ^ij^	2.89 ± 0.17 ^efg^
		‘Redbor F_1_’	10.08 ± 0.39 ^h^	1.97 ± 0.01 ^p^	2.30 ± 0.30 ^ef^	0.26 ± 0.02 ^ab^	5.24 ± 0.31 ^jk^	3.40 ± 0.23 ^hij^
	8-OH-7-I-5QSA	‘Oldenbor F_1_’	10.40 ± 0.14 ^h^	2.04 ± 0.03 ^r^	3.26 ± 0.11 ^h^	0.58 ± 0.03 ^fg^	5.18 ± 0.08 ^jk^	3.01 ± 0.01 ^efgh^
		‘Redbor F_1_’	10.35 ± 0.30 ^h^	2.20 ± 0.01 ^u^	2.72 ± 0.09 ^g^	0.34 ± 0.01 ^c^	5.63 ± 0.11 ^l^	3.68 ± 0.08 ^jk^
	5-Cl-7-I-8-Q	‘Oldenbor F_1_’	10.18 ± 0.15 ^h^	2.08 ± 0.02 ^s^	3.44 ± 0.06 ^h^	0.58 ± 0.02 ^fg^	5.11 ± 0.06 ^jk^	3.38 ± 0.13 ^ghij^
		‘Redbor F_1_’	10.05 ± 0.54 ^h^	2.22 ± 0.02 ^u^	2.83 ± 0.12 ^g^	0.46 ± 0.02 ^d^	5.36 ± 0.15 ^kl^	3.96 ± 0.17 ^k^
Steaming	Control	‘Oldenbor F_1_’	17.54 ± 0.55 ^l^	0.65 ± 0.01 ^k^	5.35 ± 0.26 ^k^	1.45 ± 0.07 ^o^	11.47 ± 0.31 ^r^	6.50 ± 0.28 ^o^
		‘Redbor F_1_’	25.38 ± 1.28 ^m^	0.51 ± 0.04 ^g^	7.45 ± 0.33 ^m^	1.19 ± 0.05 ^m^	17.56 ± 0.27 ^t^	9.14 ± 0.45 ^p^
	KIO_3_	‘Oldenbor F_1_’	14.75 ± 0.54 ^j^	0.30 ± 0.02 ^b.c^	4.47 ± 0.38 ^j^	0.97 ± 0.03 ^l^	10.34 ± 0.37 ^o^	4.76 ± 0.01 ^lm^
		‘Redbor F_1_’	15.03 ± 0.49 ^jk^	0.31 ± 0.02 ^c^	4.27 ± 0.23 ^ij^	0.92 ± 0.02 ^k^	10.68 ± 0.25 ^p^	5.14 ± 0.30 ^m^
	8-OH-7-I-5QSA	‘Oldenbor F_1_’	18.21 ± 0.96 ^l^	0.71 ± 0.04 ^l^	5.36 ± 0.24 ^k^	1.32 ± 0.07 ^n^	13.50 ± 0.22 ^s^	6.76 ± 0.72 ^o^
		‘Redbor F_1_’	15.50 ± 0.81 ^k^	0.22 ± 0.01 ^a^	4.34 ± 0.15 ^j^	0.85 ± 0.03 ^k^	11.32 ± 0.13 ^r^	6.02 ± 0.18 ^n^
	5-Cl-7-I-8-Q	‘Oldenbor F_1_’	18.19 ± 0.91 ^l^	0.52 ± 0.02 ^h^	6.06 ± 0.29 ^l^	1.22 ± 0.04 ^m^	11.59 ± 0.31 ^r^	5.89 ± 0.47 ^n^
		‘Redbor F_1_’	12.53 ± 0.50 ^i^	0.22 ± 0.02 ^a^	3.34 ± 0.12 ^h^	0.77 ± 0.03 ^i^	9.59 ± 0.11 ^n^	4.63 ± 0.20 ^l^
Blanching	Control	‘Oldenbor F_1_’	3.84 ± 0.16 ^c^	0.49 ± 0.00 ^f^	1.14 ± 0.03 ^b^	0.34 ± 0.01 ^c^	2.14 ± 0.03 ^c^	1.39 ± 0.09 ^b^
		‘Redbor F_1_’	7.44 ± 0.27 ^g^	0.89 ± 0.05 ^n^	2.03 ± 0.09 ^de^	0.55 ± 0.02 ^efg^	4.46 ± 0.11 ^h^	2.32 ± 0.13 ^c^
	KIO_3_	‘Oldenbor F_1_’	3.63 ± 0.19 ^c^	0.39 ± 0.00 ^d^	1.14 ± 0.01 ^b^	0.30 ± 0.01 ^bc^	1.96 ± 0.01 ^c^	1.00 ± 0.04 ^ab^
		‘Redbor F_1_’	3.05 ± 0.17 ^ab^	0.43 ± 0.02 ^e^	0.88 ± 0.03 ^ab^	0.22 ± 0.01 ^a^	1.67 ± 0.02 ^b^	0.89 ± 0.06 ^a^
	8-OH-7-I-5QSA	‘Oldenbor F_1_’	2.58 ± 0.10 ^a^	0.27 ± 0.01 ^bc^	0.83 ± 0.03 ^a^	0.25 ± 0.01 ^ab^	1.39 ± 0.04 ^a^	0.72 ± 0.01 ^a^
		‘Redbor F_1_’	3.48 ± 0.03 ^bc^	0.38 ± 0.02 ^d^	1.05 ± 0.03 ^a^	0.25 ± 0.00 ^ab^	1.94 ± 0.05 ^bc^	1.06 ± 0.01 ^ab^
	5-Cl-7-I-8-Q	‘Oldenbor F_1_’	3.53 ± 0.16 ^bc^	0.38 ± 0.03 ^d^	1.14 ± 0.03 ^b^	0.33 ± 0.01 ^c^	1.86 ± 0.07 ^bc^	0.92 ± 0.00 ^a^
		‘Redbor F_1_’	12.54 ± 0.62 ^i^	1.16 ± 0.03 ^o^	4.02 ± 0.20 ^i^	0.97 ± 0.04 ^l^	6.85 ± 0.26 ^m^	3.94 ± 0.02 ^k^
Boiling	Control	‘Oldenbor F_1_’	7.13 ± 0.08 ^fg^	0.75 ± 0.02 ^m^	2.29 ± 0.11 ^ef^	0.71 ± 0.03 ^h^	3.66 ± 0.13 ^ef^	2.99 ± 0.02 ^efgh^
		‘Redbor F_1_’	6.26 ± 0.11 ^e^	0.56 ± 0.00 ^ij^	2.05 ± 0.03 ^de^	0.55 ± 0.01 ^efg^	3.69 ± 0.03 ^ef^	2.82 ± 0.05 ^def^
	KIO_3_	‘Oldenbor F_1_’	7.28 ± 0.09 ^fg^	0.72 ± 0.01 ^l^	2.38 ± 0.04 ^f^	0.74 ± 0.03 ^hi^	3.91 ± 0.04 ^rg^	3.15 ± 0.04 ^fghi^
		‘Redbor F_1_’	6.66 ± 0.21 ^ef^	0.55 ± 0.01 ^i^	2.08 ± 0.05 ^de^	0.58 ± 0.03 ^fg^	3.86 ± 0.02 ^r^	2.99 ± 0.04 ^efgh^
	8-OH-7-I-5QSA	‘Oldenbor F_1_’	6.56 ± 0.04 ^ef^	0.71 ± 0.00 ^l^	2.07 ± 0.05 ^de^	0.59 ± 0.01 ^g^	3.46 ± 0.05 ^e^	2.66 ± 0.04 ^cdef^
		‘Redbor F_1_’	6.23 ± 0.14 ^e^	0.70 ± 0.01 ^l^	1.93 ± 0.08 ^cd^	0.43 ± 0.02 ^d^	3.40 ± 0.09 ^e^	2.65 ± 0.14 ^cde^
	5-Cl-7-I-8-Q	‘Oldenbor F_1_’	5.32 ± 0.30 ^d^	0.46 ± 0.02 ^f^	1.70 ± 0.03 ^c^	0.53 ± 0.03 ^ef^	2.89 ± 0.01 ^d^	2.38 ± 0.13 ^cd^
		‘Redbor F_1_’	7.25 ± 0.13 ^fg^	0.58 ± 0.01 ^j^	2.30 ± 0.03 ^ef^	0.56 ± 0.03 ^efg^	4.15 ± 0.05 ^g^	3.36 ± 0.13 ^ghij^

Results are shown as mean ± standard error (SE); n = 3; homogeneous groups refer to a three-factor analysis of variance: factor No. 1 culinary treatment: raw, steaming, blanching, boiling x factor No. 2 type of enrichment: control, KIO_3_, 8-OH-7-I-5QSA, 5-Cl-7-I-8-Q x factor No. 3 kale cultivar: ’Oldenbor F1’ and ’Redbor F1’; means followed by the same letter are not significantly different *p* < 0.05 (Duncan’s post-hoc test).

Blanching and boiling resulted in a significant decrease in dry matter content in both cultivars, in each enrichment variant, compared to the control. The boiling process caused the greatest reduction in dry matter content.

### Ash

Table 3 (S2B Fig) shows the results of ash compactness in the leaves of raw and heat-treated kale. The highest amount of ash was recorded in the raw curly leaves of kale in both cultivars. However, thermal processes such as steaming, blanching, and boiling, lowered the amount of ash content, compared to raw leaves of kale. During the boiling process, the ash content decreased up to 82%.

### Protein

The results of protein content in the leaves of raw and heat-treated kale are summarised in Table 3 (S2C Fig). The application of thermal processes significantly affected the protein content of the biofortified kale cultivars tested, compared to the control. During the steaming process, there was a significant increase in the protein content of the leaves of ‘Oldenbor F_1_’ and ‘Redbor F_1_‘ kale, in each of the applied enrichments KIO_3_, 8-OH-7-I-5QSA, and 5-Cl-7-I-8-Q. For example, kale enriched with 8-OH-7-I-5QSA during steaming increased the protein content from 3.26 to 5.36 ‘Oldenbor F_1_’ and from 2.72 to 4.34 ‘Redbor F_1_’ g∙100 g^-1^ F.W.

The opposite phenomenon was observed during the blanching and boiling processes. Kale enriched with 8-OH-7-I-5QSA during blanching decreased the protein content from 3.26 to 0.83 ‘Oldenbor F_1_’ and from 2.72 to 1.05 ‘Redbor F1’ g∙100 g^-1^ F.W. For instance, kale enriched with 8-OH-7-I-5QSA during boiling decreased the protein content from 3.26 to 2.07 ‘Oldenbor F_1_’ and from 2.72 to 1.93 ‘Redbor F1’ g∙100 g^-1^ F.W. During these both processes, there was a statistically significant decrease in the protein content of kale leaves.

### Fat

The results of fat content in the leaves of raw and heat-treated kale are shown in Table 3 (S2D Fig). In general, kale has a low fat content in its leaves.

The application of thermal processes significantly affected the fat content in the tested cultivars of biofortified kale, compared to the control. During the steaming and boiling processes, there was a significant increase in fat content in the leaves of ‘both kale cultivars, in each of the applied enrichments, KIO_3_, 8-OH-7-I-5QSA, and 5-Cl-7-I-8-Q, in comparison to the control. For example, kale enriched with 8-OH-7-I-5QSA during steaming increased the fat content from 0.58 to 1.32 ‘Oldenbor F_1_’ and from 0.46 to 0.85 ‘Redbor F_1_’ g∙100 g^-1^ F.W. Kale enriched with KIO_3_ during boiling increased the fat content from 0.52 to 0.74 ‘Oldenbor F_1_’ and from 0.26 to 0.58 ‘Redbor F_1_’ g∙100 g^-1^ F.W. Only during blanching did the fat content decrease, e.g. kale enriched with 8-OH-7-I-5QSA from 0.58 to 0.25 ‘Oldenbor F_1_’ and from 0.46 to 0.25 ‘Redbor F_1_’ g∙100 g^-1^ F.W. However, the situation was not the same in every case.

### Total carbohydrate

Table 3 (S2E Fig) shows the results of total carbohydrate content in the leaves of raw and heat-treated kale. The application of thermal processes significantly affected the total carbohydrate content of the tested cultivars of the biofortified kale, compared to the control. During the steaming process, there was a significant increase in the total carbohydrate content of ‘Oldenbor F_1_’ and ‘Redbor F_1_‘ kale leaves, in each of the applied enrichments, KIO_3_, 8-OH-7-I-5QSA, and 5-Cl-7-I-8-Q, relative to the control. For instance, kale enriched with 8-OH-7-I-5QSA during steaming increased the total carbohydrate content from 5.18 to 13.50 ‘Oldenbor F_1_’ and from 5.63 to 11.32 ‘Redbor F_1_’ g∙100 g^-1^ F.W.

After applying the blanching and boiling processes, there was a significant reduction in total carbohydrate content. For example, kale enriched with 8-OH-7-I-5QSA during blanching decreased the total carbohydrate content from 5.18 to 1.3 ‘Oldenbor F_1_’ and from 5.63 to 1.94 ‘Redbor F_1_’ g∙100 g^-1^ F.W.

### Dietary fibre

The results of dietary fibre content analysis in the leaves of raw and heat-treated kale are shown in Table 3 (S2F Fig). The application of thermal processes significantly affected the fibre content of the biofortified kale cultivars tested, compared to the control. During the steaming process, there was a significant increase in the fibre content of ‘Oldenbor F_1_‘ and ‘Redbor F_1_‘ kale leaves, in each of the applied enrichments, KIO_3_, 8-OH-7-I-5QSA, and 5-Cl-7-I-8-Q, relative to the control. For example, kale enriched with 8-OH-7-I-5QSA during steaming increased the dietary fibre content from 3.01 to 6.76 ‘Oldenbor F_1_’ and from 3.68 to 6.02 ‘Redbor F_1_’ g∙100 g^-1^ F.W.

After blanching and boiling processes, there was a significant reduction in the fibre content. For instance, kale enriched with 8-OH-7-I-5QSA during blanching decreased the dietary fibre content from 3.01 to 0.72 ‘Oldenbor F_1_’ and from 3.68 to 1.06 ‘Redbor F_1_’ g∙100 g^-1^ F.W.

## The influence of thermal processes on L-ascorbic acid, total carotenoids, total phenolics content and free radical quenching capacity of ABTS^●+^ concentration in kale

### Total phenolics

The phenolics content of kale raw and after the cooking processes are shown in Table 4 (S3A Fig). Of particular note is that biofortification with KIO_3_ and iodoquinolines in the form of 8-OH-7-I-5QSA and 5-Cl-7-I-8-Q statistically significantly improved the polyphenol profile of the raw kale, relative to the control raw kale. Total polyphenol content of control kale was 547.71 mg (‘Oldenbor F_1_’) and 507.40 (‘Redbor F_1_’), while the most higer of all 8-OH-7-I-5QSA was 575.27 (‘Oldenbor F_1_’) and 610.89 (‘Redbor F_1_’), mg GAE∙100 g^-1^ F.W., respectively.

**Table 4:** The antioxidant activity and content of total phenolics, total carotenoids, and ascorbic acid in kale ‘Oldenbor F_1_’ and ‘Redbor F_1_‘ before and after heat treatments.

Culinary treatment	Enrichment	Cultivar	Total Phenolics mg GAE·100 g^−1^ F.W.	ABTS^●+^ μmol Trolox·g^−1^ F.W.	Ascorbic Acid mg·100 g^−1^ F.W.	Total Carotenoids mg·100 g^−1^ F.W.
Raw	Control	‘Oldenbor F_1_’	547.71 ± 4.65 ^s^	17.93 ± 0.67 ^l^	284.97 ± 14.23 ^gh^	17.46 ± 0.75 ^defgh^
		‘Redbor F_1_’	507.40 ± 13.72 ^r^	16.57 ± 0.81 ^jk^	237.01 ± 53.17 ^fg^	24.00 ± 2.39 ^lmn^
	KIO_3_	‘Oldenbor F_1_’	571.24 ± 19.79 ^t^	17.03 ± 0.55 ^jkl^	181.56 ± 7.94 ^def^	17.83 ± 4.57 ^efgh^
		‘Redbor F_1_’	532.93 ± 14.16 ^s^	20.10 ± 0.89 ^m^	334.42 ± 43.57 ^h^	19.19 ± 1.85 ^fghi^
	8-OH-7-I-5QSA	‘Oldenbor F_1_’	575.27 ± 2.33 ^t^	17.23 ± 0.90 ^kl^	189.73 ± 69.11 ^def^	20.48 ± 2.47 ^hijk^
		‘Redbor F_1_’	610.89 ± 28.23 ^u^	17.27 ± 0.87 ^kl^	240.29 ± 92.65 ^fg^	19.87 ± 1.20 ^ghi^
	5-Cl-7-I-8-Q	‘Oldenbor F_1_’	538.31 ± 8.79 ^s^	19.47 ± 0.32 ^m^	232.11 ± 30.12 ^efg^	19.87 ± 2.57 ^ghij^
		‘Redbor F_1_’	575.94 ± 18.73 ^t^	21.43 ± 1.53 ^n^	174.61 ± 85.00 ^de^	20.27 ± 1.88 ^hij^
Steaming	Control	‘Oldenbor F_1_’	365.38 ± 14.71 ^o^	8.27 ± 0.40 ^e^	179.89 ± 6.66 ^def^	23.27 ± 1.27 ^jklm^
		‘Redbor F_1_’	292.15 ± 12.81 ^k^	16.07 ± 0.35 ^i^	65.67 ± 14.59 ^abc^	23.66 ± 0.32 ^klm^
	KIO_3_	‘Oldenbor F_1_’	358.60 ± 13.38 ^no^	16.97 ± 0.15 ^jkl^	153.89 ± 38.73 ^d^	31.41 ± 0.89 ^o^
		‘Redbor F_1_’	312.47 ± 5.54 ^l^	13.74 ± 0.72 ^h^	86.62 ± 44.45 ^c^	24.48 ± 0.87 ^lmn^
	8-OH-7-I-5QSA	‘Oldenbor F_1_’	342.47 ± 13.71 ^mn^	14.17 ± 0.31 ^h^	142.83 ± 37.91 ^d^	23.75 ± 0.04 ^klm^
		‘Redbor F_1_’	328.82 ± 9.99 ^lm^	12.47 ± 0.31 ^g^	71.53 ± 17.75 ^bc^	27.24 ± 0.21 ^n^
	5-Cl-7-I-8-Q	‘Oldenbor F_1_’	439.03 ± 1.48 ^p^	17.87 ± 0.50 ^l^	171.19 ± 24.41 ^de^	24.15 ± 0.55 ^lmn^
		‘Redbor F_1_’	376.13 ± 19.28 ^o^	12.31 ± 0.60 ^g^	145.26 ± 50.03 ^d^	26.16 ± 1.12 ^mn^
Blanching	Control	‘Oldenbor F_1_’	183.87 ± 2.96 ^fg^	9.40 ± 0.46 ^f^	41.46 ± 3.20 ^ab^	16.52 ± 1.37 ^cdefg^
		‘Redbor F_1_’	199.03 ± 2.87 ^gh^	13.68 ± 0.73 ^h^	15.41 ± 3.35 ^ab^	18.37 ± 2.35 ^efgh^
	KIO_3_	‘Oldenbor F_1_’	152.26 ± 4.75 ^cd^	8.38 ± 0.17 ^e^	19.06 ± 4.89 ^ab^	16.13 ± 1.41 ^bcde^
		‘Redbor F_1_’	244.62 ± 4.29 ^j^	16.68 ± 0.51 ^jk^	37.42 ± 1.70 ^abc^	24.13 ± 1.09 ^lmn^
	8-OH-7-I-5QSA	‘Oldenbor F_1_’	204.95 ± 3.13 ^hi^	9.48 ± 0.66 ^f^	24.03 ± 12.24 ^abc^	16.66 ± 0.79 ^cdefg^
		‘Redbor F_1_’	217.74 ± 6.83 ^i^	15.52 ± 0.75 ^i^	24.96 ± 2.30 ^abc^	15.66 ± 0.81 ^bcde^
	5-Cl-7-I-8-Q	‘Oldenbor F_1_’	206.66 ± 3.36 ^hi^	9.74 ± 0.12 ^f^	25.40 ± 16.96 ^abc^	20.16 ± 0.58 ^hij^
		‘Redbor F_1_’	179.14 ± 2.99 ^ef^	11.92 ± 0.73 ^g^	21.46 ± 2.06 ^ab^	22.56 ± 0.92 ^ijkl^
Boiling	Control	‘Oldenbor F_1_’	154.30 ± 5.22 ^cd^	2.82 ± 0.09 ^ab^	15.71 ± 3.43 ^ab^	13.23 ± 2.41 ^abc^
		‘Redbor F_1_’	140.32 ± 1.29 ^bc^	2.26 ± 0.09 ^a^	10.25 ± 2.18 ^ab^	14.22 ± 2.04 ^bcd^
	KIO_3_	‘Oldenbor F_1_’	166.02 ± 1.62 ^def^	6.84 ± 0.25 ^d^	21.31 ± 4.00 ^ab^	14.31 ± 1.49 ^bcd^
		‘Redbor F_1_’	125.48 ± 4.03 ^ab^	3.16 ± 0.09 ^ab^	0.00 ± 0.00 ^a^	12.96 ± 3.61 ^ab^
	8-OH-7-I-5QSA	‘Oldenbor F_1_’	129.36 ± 5.33 ^ab^	3.30 ± 0.18 ^ab^	17.79 ± 0.20 ^ab^	13.52 ± 3.01 ^abc^
		‘Redbor F_1_’	118.39 ± 0.65 ^a^	4.34 ± 0.27 ^c^	20.73 ± 3.73 ^ab^	10.89 ± 1.20 ^a^
	5-Cl-7-I-8-Q	‘Oldenbor F_1_’	139.35 ± 2.59 ^bc^	2.34 ± 0.09 ^a^	0.00 ± 0.00 ^a^	10.59 ± 1.42 ^a^
		‘Redbor F_1_’	164.41 ± 4.40 ^de^	3.44 ± 0.18 ^bc^	11.81 ± 1.25 ^ab^	15.27 ± 1.83 ^bcde^

Results are shown as mean ± standard error (SE); n = 3; homogeneous groups refer to a three-factor analysis of variance: factor No. 1 culinary treatment: raw, steaming, blanching, boiling x factor No. 2 type of enrichment: control, KIO_3_, 8-OH-7-I-5QSA, 5-Cl-7-I-8-Q x factor No. 3 kale cultivar: ’Oldenbor F_1_’ and ’Redbor F_1_’; means followed by the same letter are not significantly different *p* < 0.05 (Duncan’s post-hoc test).

The boiling processes had a statistically significant effect on the phenolics content of the kale extracts tested, relative to the control. The steaming process had the least effect on reducing the phenolics content. The next process was blanching. The boiling process had the greatest effect on the degradation and leaching of polyphenols from kale.

### Free radical quenching capacity of ABTS^●+^

The results of ABTS^●+^ radical activity in kale raw and after the cooking processes are presented in Table 4 (S3B Fig). Of particular note is that biofortification with KIO3 and iodoquinolines in the form of 8-OH-7-I-5QSA and 5-Cl-7-I-8-Q statistically significantly improved the antioxidant activity of raw kale, relative to the control. The biofortification process had a positive effect on improving the antioxidant activity of the kale tested. Perhaps this is related to the higher proportion of iodine.

The heat processes had a statistically significant effect on the antioxidant activity in the kale extracts tested, compared to the control raw kale. The steaming and blanching processes increased the antioxidant activity in kale, relative to the control raw kale. Only the boiling process had the greatest effect on the degradation and significant reduction in the antioxidant activity of the kale leaves.

### L-ascorbic acid

The content of L-ascorbic acid in kale raw and after the cooking processes are shown in Table 4 (S3C Fig). Vitamin C, also known as ascorbic acid, is a water-soluble vitamin with high sensitivity to heat, light, and oxygen. The analyses show that the green cultivar ‘Oldenbor F_1_‘showed a significantly higher L-ascorbic acid content (285 mg∙100 g^-1^ F.W.) than the red cultivar Redbor F_1_‘ (237 mg∙100 g^-1^ F.W.). The biofortification process did not significantly improve or reduce L-ascorbic acid in raw kale leaves, not subjected to heat treatment.

The heat processes had a statistically significant effect on the L-ascorbic acid content of kale, relative to the control. The steaming process had the least effect on reducing the L-ascorbic acid content. The blanching and boiling processes significantly affected the reduction through oxidation of L-ascorbic acid in kale leaves. However, blanching had less effect on reducing the L-ascorbic acid content than boiling.

### Total carotenoids

The total carotenoids content of kale raw and after the cooking processes are shown in Table 4 (S3D Fig). Biofortification with KIO_3_ and 5-Cl-7-I-8-Q, significantly improved the carotenoid content of raw kale, compared to the control. The steaming processes significantly increased carotenoids in leaves of kale non- and biofortified with iodine. On the other hand, the blanching and boiling processes significantly lower carotenoids in leaves of kale of both cultivars ’Oldenbor F_1_’ and ’Redbor F_1_’.

### The results of thermal processes on assessment of iodine-biofortified kale for consumer health safety

The biofortification of kale leaves with iodine compounds increased the participation of the recommended daily intake of iodine (RDA-I) -and HQ coefficient value through the consumption of 50 and 100 g portions of kale leaves by adults (Table 5, S4 Fig). The hazard quotient (HQ) value is intended to inform consumers about the safety of food consumption. If the HQ value is higher than 1, adverse health effects are possible. The HQ value for the consumption of raw kale leaves not subjected to any boiling process, both ‘Oldenbor F_1_‘ and ‘Redbor F_1_‘ cultivars, ranged from 0.01 to 0.17 (for a 50 g portion, respectively) and from 0.03 to 0.34 (for a 100 g portion, respectively), representing a harmless consumption for consumers. The highest statistically significant RDA-I (calculated for 50 g and 100 g portion) was observed in kale leaves that had been biofortified with KIO_3_ and 8-OH-7-I-5QSA, relative to the control. The biofortification with 5-Cl-7-I-8-Q was slightly weaker but continued to be statistically significant. The recommended daily intake of iodine for adults is 150 μg [42]. An average serving of 50 g of iodine biofortified kale, not subjected to a culinary process, used in a salad or smoothie consumed daily, could cover 76% (KIO_3_), 76% (8-OH-7-I-5QSA), 37% (5-Cl-7-I-8-Q) of RDA-I, compared to the non-biofortified control kale, which covers only 6.4% of iodine requirements (% calculated from the average of both cultivars). Heat treatment reduced the % RDA I of 50 g portion of the control (non-biofortified) kale by 50% during steaming, 80% blanching and 78% boiling, respectively. Similarly, heat treatment of kale biofortified with KIO_3_ reduced % RDA I of a 50 g portion by 19% during steaming, 56% blanching, 67% boiling. Correspondingly, in 8-OH-7-I-5QSA, the % RDA I of a 50 g serving of kale decreased by 2.5% during steaming, 39% blanching, 55% boiling. In kale biofortified with 5-Cl-7-I-8-Q, there was a reduction in % RDA I of a 50 g portion by 11% during steaming, 59% during blanching 47% during boiling.

**Table 5:** Percentage coverage of Recommended Daily Allowance for iodine (% RDA-I) and hazard quotient (HQ) for intake of iodine through consumption of 100 g and 50 g portions of kale ‘Oldenbor F_1_‘ and ‘Redbor F_1_‘ before and after heat treatment in individual by adults 70 kg body weight.

Culinary treatment	Enrichment	Cultivar	Daily intake of I with 50 g of kale (μg I·day^−1^)	Daily intake of I with 100 g of kale (μg I·day^−1^)	% RDA I (in 50 g portion of kale)	% RDA I (in 100 g portion of kale)	HQ for 50 g portion of kale	HQ for 100 g portion of kale
Raw	Control	‘Oldenbor F_1_’	8.93 ± 1.18 ^d^	17.85 ± 2.37 ^d^	5.95 ± 0.79 ^d^	11.90 ± 1.58 ^d^	0.012 ± 0.00 ^b^	0.024 ± 0.01 ^bc^
		‘Redbor F_1_’	10.23 ± 0.54 ^d^	20.45 ± 1.09 ^d^	6.82 ± 0.36 ^d^	13.63 ± 0.72 ^d^	0.013 ± 0.00 ^b^	0.026 ± 0.01 ^bc^
	KIO_3_	‘Oldenbor F_1_’	122.13 ± 0.36 ^t^	244.26 ± 0.72 ^t^	81.42 ± 0.24 ^t^	162.84 ± 0.48 ^t^	0.167± 0.00 ^m^	0.333 ± 0.00 ^o^
		‘Redbor F_1_’	106.55 ± 1.04 ^r^	213.10 ± 2.07 ^r^	71.03 ± 0.69 ^r^	142.07 ± 1.38 ^r^	0.131 ± 0.00 ^j^	0.263 ± 0.00 ^m^
	8-OH-7-I-5QSA	‘Oldenbor F_1_’	104.90 ± 1.88 ^r^	209.80 ± 3.77 ^r^	69.93 ± 1.26 ^r^	139.87 ± 2.51 ^r^	0.144 ± 0.01 ^k^	0.289 ± 0.01 ^n^
		‘Redbor F_1_’	121.74 ± 4.11 ^t^	243.47 ± 8.21 ^t^	81.16 ± 2.74 ^t^	162.31 ± 5.48 ^t^	0.168 ± 0.01 ^l^	0.335 ± 0.01 ^o^
	5-Cl-7-I-8-Q	‘Oldenbor F_1_’	60.30 ± 2.87 ^k^	120.60 ± 5.75 ^k^	40.20 ± 1.92 ^k^	80.40 ± 3.83 ^k^	0.081 ± 0.00 ^h^	0.161 ± 0.01 ^k^
		‘Redbor F_1_’	51.70 ± 0.91 ^j^	103.40 ± 1.82 ^j^	34.47 ± 0.60 ^j^	68.93 ± 1.21 ^j^	0.071 ± 0.00 ^g^	0.142 ± 0.01 ^j^
Steaming	Control	‘Oldenbor F_1_’	5.16 ± 0.17 ^c^	10.33 ± 0.33 ^c^	3.44 ± 0.11 ^c^	6.88 ± 0.22 ^c^	0.012 ± 0.00 ^b^	0.024 ± 0.00 ^b^
		‘Redbor F_1_’	4.46 ± 0.09 ^c^	8.91 ± 0.17 ^bc^	2.97 ± 0.06 ^bc^	5.94 ± 0.12 ^bc^	0.015 ± 0.01 ^b^	0.029 ± 0.00 ^c^
	KIO_3_	‘Oldenbor F_1_’	95.20 ± 4.13 ^o^	190.40 ± 8.25 ^o^	63.47 ± 2.75 ^o^	126.93 ± 5.50 ^o^	0.182 ± 0.01 ^o^	0.365 ± 0.02 ^r^
		‘Redbor F_1_’	89.69 ± 0.91 ^n^	179.39 ± 1.82 ^n^	59.80 ± 0.61 ^n^	119.59 ± 1.21 ^n^	0.175 ± 0.00 ^n^	0.350 ± 0.01 ^p^
	8-OH-7-I-5QSA	‘Oldenbor F_1_’	101.58 ± 1.48 ^p^	203.16 ± 2.96 ^p^	67.72 ± 0.99 ^p^	135.44 ± 1.97 ^p^	0.240 ± 0.00 ^p^	0.481 ± 0.01 ^s^
		‘Redbor F_1_’	119.40 ± 1.05 ^s^	238.80 ± 2.09 ^s^	79.60 ± 0.70 ^s^	159.20 ± 1.39 ^s^	0.240 ± 0.00 ^p^	0.481 ± 0.01 ^s^
	5-Cl-7-I-8-Q	‘Oldenbor F_1_’	49.37 ± 0.33 ^j^	98.73 ± 0.66 ^j^	32.91 ± 0.22 ^j^	65.82 ± 0.44 ^j^	0.117 ± 0.00 ^i^	0.233 ± 0.00 ^l^
		‘Redbor F_1_’	49.92 ± 1.58 ^j^	99.84 ± 3.16 ^j^	33.28 ± 1.05 ^j^	66.56 ± 2.11 ^j^	0.081 ± 0.00 ^h^	0.162 ± 0.01 ^k^
Blanching	Control	‘Oldenbor F_1_’	2.17 ± 0.05 ^ab^	4.34 ± 0.10 ^ab^	1.45 ± 0.04 ^ab^	2.90 ± 0.07 ^ab^	0.001 ± 0.00 ^a^	0.002 ± 0.00 ^a^
		‘Redbor F_1_’	1.67 ± 0.07 ^a^	3.34 ± 0.13 ^a^	1.12 ± 0.05 ^a^	2.23 ± 0.09 ^a^	0.002 ± 0.00 ^a^	0.003 ± 0.00 ^a^
	KIO_3_	‘Oldenbor F_1_’	38.97 ± 1.79 ^h^	77.94 ± 3.59 ^h^	25.98 ± 1.20 ^h^	51.96 ± 2.39 ^h^	0.018 ± 0.00 ^c^	0.037 ± 0.00 ^d^
		‘Redbor F_1_’	62.15 ± 0.78 ^kl^	124.30 ± 1.56 ^kl^	41.43 ± 0.52 ^kl^	82.86 ± 1.04 ^kl^	0.025 ± 0.00 ^c^	0.049 ± 0.00 ^e^
	8-OH-7-I-5QSA	‘Oldenbor F_1_’	68.87 ± 1.31 ^m^	137.74 ± 2.63 ^m^	45.91 ± 0.88 ^m^	91.82 ± 1.75 ^m^	0.023 ± 0.00 ^c^	0.046 ± 0.00 ^e^
		‘Redbor F_1_’	69.89 ± 2.27 ^m^	139.78 ± 4.53 ^m^	46.60 ± 1.51 ^m^	93.19 ± 3.03 ^m^	0.032 ± 0.00 ^d^	0.063 ± 0.01 ^g^
	5-Cl-7-I-8-Q	‘Oldenbor F_1_’	28.11 ± 0.66 ^f^	56.22 ± 1.32 ^f^	18.74 ± 0.44 ^f^	37.48 ± 0.88 ^f^	0.013 ± 0.00 ^b^	0.026 ± 0.01 ^bc^
		‘Redbor F_1_’	16.58 ± 0.41 ^e^	33.16 ± 0.82 ^e^	11.06 ± 0.28 ^e^	22.11 ± 0.54 ^e^	0.027 ± 0.00 ^d^	0.054 ± 0.01 ^ef^
Boiling	Control	‘Oldenbor F_1_’	2.60 ± 0.04 ^ab^	5.19 ± 0.08 ^ab^	1.73 ± 0.03 ^ab^	3.46 ± 0.05 ^ab^	0.002 ± 0.00 ^a^	0.005 ± 0.00 ^a^
		‘Redbor F_1_’	1.54 ± 0.05 ^a^	3.08 ± 0.10 ^a^	1.03 ± 0.03 ^a^	2.05 ± 0.07 ^a^	0.001 ± 0.00 ^a^	0.003 ± 0.00 ^a^
	KIO_3_	‘Oldenbor F_1_’	42.11 ± 0.43 ^i^	84.21 ± 0.86 ^i^	28.07 ± 0.28 ^i^	56.14 ± 0.57 ^i^	0.040 ± 0.00 ^e^	0.080 ± 0.00 ^h^
		‘Redbor F_1_’	32.94 ± 0.80 ^g^	65.88 ± 1.60 ^g^	21.96 ± 0.54 ^g^	43.92 ± 1.07 ^g^	0.028 ± 0.00 ^d^	0.057 ± 0.00 ^fg^
	8-OH-7-I-5QSA	‘Oldenbor F_1_’	37.30 ± 0.18 ^h^	74.60 ± 0.36 ^h^	24.87 ± 0.12 ^h^	49.73 ± 0.24 ^h^	0.032 ± 0.00 ^d^	0.064 ± 0.00 ^fg^
		‘Redbor F_1_’	64.39 ± 3.53 ^l^	128.78 ± 7.05 ^l^	42.93 ± 2.35 ^l^	85.85 ± 4.70 ^l^	0.052 ± 0.01 ^f^	0.104 ± 0.01 ^g^
	5-Cl-7-I-8-Q	‘Oldenbor F_1_’	29.69 ± 1.55 ^f^	59.38 ± 3.10 ^f^	19.79 ± 1.03 ^f^	39.58 ± 2.06 ^f^	0.021 ± 0.00 ^c^	0.041 ± 0.00 ^d^
		‘Redbor F_1_’	14.97 ± 0.21 ^e^	29.93 ± 0.43 ^e^	9.98 ± 0.14 ^e^	19.96 ± 0.28 ^e^	0.014 ± 0.00 ^b^	0.028 ± 0.00 ^c^

Results are shown as mean ± standard error (SE); n = 4; homogeneous groups refer to a three-factor analysis of variance: factor No. 1 culinary treatment: raw, steaming, blanching, boiling x factor No. 2 type of enrichment: control, KIO_3_, 8-OH-7-I-5QSA, 5-Cl-7-I-8-Q x factor No. 3 kale cultivar ’Oldenbor F_1_’ and ’Redbor F_1_’ (3); means followed by the same letter are not significantly different *p* < 0.05 (Duncan’s post-hoc test).

The intake of I KIO_3_, 8-OH-7-I-5QSA, and 5-Cl-7-I-8-Q is not lower than the tolerable upper intake level for iodine, which is 1100 μg per day^-1^. It is noteworthy that the boiling processes significantly influenced the iodine concentration and thus the %RDA-I and HQ values. The values of %RDA and HQ decreased according to the decrease in iodine concentration during steaming, blanching, and boiling processes.

## Discussion

The heat treatment of food induces a number of biological, physical and chemical changes. The cooking process has a huge impact on the health-promoting bioactive compounds and basic composition of *Brassica* plants. Based on the statistical analyses, it was demonstrated that the process of biofortification of kale leaves with iodoquinolines influenced of the higher amount of iodine compared to control. Iodine is acknowledged as an essential micronutrient for the human body. Its insufficiency can cause many pathologies, including physical and mental disorders [1].

### Safety of consumption of biofortified kale

Biofortification of plants can be an effective strategy to reduce micronutrient malnutrition worldwide. In some cases, raw biofortified kale may allow consumption of more iodine than recommended, but excessive regular consumption of iodine with a biofortified vegetable such as kale seems impossible.

Depending on the kind of biofortification method used i.e., foliar, soil, hydroponic, iodine is accumulated in the plant to varying degrees. The form of iodine (inorganic/organic) is also of great importance. Based on current reports, leafy vegetables, including kale [45], accumulate iodine best when using hydroponic biofortification. The higher the accumulation of iodine in the raw vegetable, the lower the negative impact of the investigated subsequent cooking process. Thus, the main purpose of biofortification is preserved, even when exposed to high temperatures and water.

In a study by Krawczyk et al. (2024) [31], biofortification of kale of two cultivars ’Oldenbor F1’ and ’Redbor F1’ with iodoquinolines in three different forms was attempted. Two years of hydroponic cultivation of kale significantly increased the total iodine content and the concentration of these compounds in the leaves. The HQ values indicated that the iodine content of kale was safe for the consumer. The results were only presented in raw kale. The chemical composition of kale was dependent on the kale variety and not on the type of compound used for iodine enrichment. No negative effect of biofortification with iodoquinolines was observed on plant growth and chemical composition (content: macro- and micronutrients, sugars: glucose, fructose, sucrose, and their sum).

Our study analysed the effect of the biofortification of kale in annual crops. The same species varieties were investigated, but only two iodoquinolines. Our main aim in this study was to investigate the influence of selected cooking processes complementing our previously obtained results (2024) [31]. However, based on two independent studies, it can be concluded that these treatments were safe for the plants tested and, in addition, improved certain nutritional values. Iodoquinolines as new compounds tested were not toxic to kale leaves.

The effects of thermal processes on the iodine content, basic composition, and antioxidant properties of biofortified kale were investigated by Waśniowska et al. (2023) [32] This study confirmed that kale biofortified with iodoquinoline (a different type of iodoquinoline tested than in our study) was exposed to iodine loss, changes in the basic composition (protein, fat, fibre, ash), and a reduction in the antioxidant properties of kale during cooking processes. This study corresponds to the changes in the properties of kale biofortified with iodoquinolines that occurred in our study, although different iodoquinoline compounds were tested.

### Iodine

The nutrient and health-promoting compounds in kale are significantly affected by traditional cooking. The use of thermal processes resulted in lower iodine concentrations in kale leaves in the control, as well as in kale biofortified with various iodine compounds. Steaming had the least effect on iodine loss. In the study of Kapusta-Duch et al. (2017) [46], the thermal processes resulted in a lower concentration of iodine in the control carrot as well as in carrots biofortified with iodine. In addition, peeling carrots caused a higher loss of iodine in the control and the biofortified carrots cooked or steamed for different times. Kiferle et al. [47] reported that the cooking process also caused losses of iodine in unpeeled and peeled tomato. Salau et al. [48] reported that losses of iodine from plant food depend not only on the time of cooking but also on the form of the plant product after its processing (e.g. pulp, final meal). On the other hand, the study by Cerratini et al. [49] evaluated the stability of iodine derived from biofortified potatoes in the preparation process of some Italian dishes. The results of these authors showed a good stability of iodine in cooking processes, also confirming the results of Comandini et al. [50].

In the study of Gonnella et al. [51], four *Brassica* vegetables (broccoli rabe, curly kale, mizuna, red mustard) were hydroponically grown with three I-IO3− rates (0, 0.75 and 1.5 mg I·L^-1^) to produce iodine-biofortified vegetables. After 21 day-iodine biofortification, the iodine accumulation in the edible raw kale reached values of 12.73 and 13.98 μg·100 g^-1^ I F.W., respectively, with 0.75 and 1.5 mg I·L^-1^. After 43 days after iodine biofortification the content of iodine wase 39.23 and 68.13 μg I·100 g^-1^ F.W., respectively. In our experiment, after 63 days of biofortification, raw kale with KIO_3_ accumulated the most iodine 244.25 ug·100 g^-1^ F.W. (‘Oldenbor F_1_’) and 213.09 ug·100 g^-1^ F.W. (‘Redbor F_1_’), respectively. The authors reported that in kale and broccoli rabe, steaming did not change iodine content at the application of 0.75 mg I·L^-1^ but doubled it in kale at 1.5 mg I·L^-1^. Four minutes of boiling always reduced iodine content, compared to the raw vegetables (in some cases more than halved), except for kale biofortified with 1.5 mg I·L^-1^, and mizuna and red mustard treated with 0.75 mg I·L^-1^ [51]. In our study, none of the culinary treatment increased the iodine content of kale. Each culinary treatment such as steaming, blanching and boiling significantly decreased the iodine content of with or without biofortified raw kale with different iodine compounds.

### Basic chemical composition

Based on analyses of the basic leaf composition of kale, the average nutrient content was confirmed to be similar to the USDA tables, according to which, 100 g of raw leaves of kale provide 1.54 g of ash, 10 g of dry matter, 2.9 g of protein, 4.4 g of carbohydrates, 4.1 g of fibre and only 1.49 g of fat [11]. The average results obtained in this work are fairly close to the values found by other authors. According to the literature, dry matter content in raw kale fluctuates broadly from 10.4 to 21.19 g/100 g fresh vegetable [52–55]. In our experiment, the biofortification process of both kale cultivars with KIO_3_, 8-OH-7-I-5QSA, 5-Cl-7-I-8-Q had no effect on changes in dry matter content, compared to the control (without biofortification). Total protein content in raw kale ranges broadly from 2.4 to 9.6 g/100 g of the vegetable [47, 48, 54, 56, 57]. Protein in kale is regarded as the high grade protein due to large amounts of essential amino acids. In this work, the average fat content in the examined raw kale was minimal which does not correspond exactly to the results obtained by Sikora and Bodziarczyk [54] and Skąpski and Dąbrowska [58]. Ash contents reported by other authors were within the range 1.1–2.2 g [49, 50, 54, 58, 59]. The average results obtained in this study were in this range. *Brassica* vegetables are an excellent source of dietary fibre. In kale, the proportion of non-digestible carbohydrates is significant and ranges from 0.8 to 3.8% [57]. The average dietary fibre content in the examined raw kale was 2.69 g/100 g fresh vegetable weight, which agrees with the findings of another authors (1.94–9.56 g/100 g) [54, 59, 60]. In the case of the effect of the biofortification process on changes in ash, protein, fat and dietary fibre content, the matter was inconclusive. In several cases, the process reduced the above components, while in others, there was a significant increase in the proportion of components. These changes varied according to the type of enrichment compound used KIO_3_, 8-OH-7-I-5QSA, 5-Cl-7-I-8-Q and the cultivar ’Oldenbor F_1_’ and ’Redbor F_1_’. The percentage of total carbohydrates in fresh kale varies between 2.4–10% [54, 59, 60]. The average scores calculated in this study were within this range. Our research shows, that it was observed the biofortification process with KIO_3_, 8-OH-7-I-5QSA, 5-Cl-7-I-8-Q increased the total carbohydrate content in each case. At the same time, improving the nutritional value of the kale of both cultivars ’Oldenbor F_1_’ and ’Redbor F_1_’.

Blanching is a heat treatment process in which food is briefly boiled or dropped into boiling water and then rapidly cooled by immersion in cold water or ice. This process aims to deactivate enzymes that can cause loss of quality and nutritional value in food and increase the shelf life of the product. However, some loss of nutrients can be experienced during blanching. Cooking vegetables usually results in more nutrient loss. Considering the different cooking techniques, steaming is one of the best ways to preserve the nutritional value. Vegetables are not immersed in water, which minimises nutrient loss, and the short processing time prevents excessive nutrient loss [61, 62], which coincides with the results obtained in this work.

A decrease in dry matter, ash, protein, fat, carbohydrates and dietary fibre content due to heat treatments (blanching and conventional boiling) in an aqueous environment may result from extraction of soluble components to water and/or absorption of the water by tissues as well as leaving some water on the surface of raw material, particularly if the surface is uneven and undulated. On the other hand, the increase of dry matter, protein, fat, carbohydrates and dietary fibre content was observed during steaming, probably because of the loss of water from the tissue and the contraction of the raw material. In the study of Florkiewicz et al. [63] and Volden et al. [64], boiling of fresh *Brassica* vegetables caused a significant decrease of the dry matter level. On the other hand, in the paper of Gębczyński and Kmiecik [65, 66], the increase of dry matter content was observed during boiling. Kapusta-Duch et al. [67] observed that the process of boiling led to statistically significant reductions in the dry matter and fat content, of 25.0 and 10.6%, respectively, compared to the raw vegetable. In parallel, the same process resulted in statistically significant increases in protein (13.4%), ash (25.8%) and dietary fibre (2.27%), compared to the vegetable before processing. In this study, the use of thermal processes significantly affected the ash content of the biofortified kale cultivars tested, compared to the control. This is likely due to the loss of macronutrients and minerals in water. During boiling processes large amounts of water can evaporate from food products. This water contains dissolved minerals and compounds that can contribute to the formation of ash. The loss of water during boiling can lead to the concentration of minerals and compounds in the remaining product, which can reduce the volume of ash. Cooking processes can lead also to denaturation and decomposition of organic components in the product. As organic components are burned or decomposed, less and less organic material remains, which can affect the amount of ash remaining in the product. During cooking processes, chemical reactions can occur between food components, including minerals. These reactions can lead to the formation of new compounds or to the oxidation of minerals, which can reduce the amount of ash [20]. Słupski et al. [68] reported that technological and culinary processing caused a significant increase in protein content. During evaporation, food products can lose excess water. This can concentrate the protein content in the rest of the product, giving the impression that the protein has "increased," although in reality the amount of protein remains the same. On the other hand, Lisiewska et al. [69] showed a 14% loss of protein during boiling of Brussels sprouts, while the losses in protein content found by Czapski [70] and Florkiewicz et al. [63] during boiling broccoli and cauliflower were of 15.6% and 10.5%, respectively. During boiling processes proteins can become denatured. Denaturation is a process in which the tertiary structure of a protein is altered, which can lead to the loosening and elongation of polypeptide chains. As a result of this process, proteins may shrink, which can lead to an apparent loss of protein volume and mass. Denaturation of proteins can result in the loss of their biological functions.

### L-ascorbic acid

The control kale cultivars tested contained an average of 261 mg of vitamin C per 100 g fresh weight of the vegetable. Other researchers obtained a half of the same results, that is Kapusta-Duch et al. [67] 108.5 mg, Korus [71] 102 mg, Korus and Lisiewska [72] 112.1 mg/100 g fresh weight of the vegetable. According to the team of Kunachowicz [57], the content of vitamin C in kale is also less than that obtained in this study, that is 120 mg/100 g fresh weight of the vegetable. Much lower results in 100 g of fresh vegetable have been reported by Pfendt et al. [73] (92.6 mg), Becerra-Moreno et al. [74] (23.78–57.26 mg) or Sikora and Bodziarczyk [54] (52.25–77.91 mg). The results presented above were for kale that had not been biofortified. Analyzing the results of our study, it can be observed that the application of KIO_3_, 8-OH-7-I-5QSA, 5-Cl-7-I-8-Q, had different effects on L-ascrobic acid content. In one case, kale of cultivar ‘Redbor F_1_’ biofortified with KIO_3_ increased the L-ascorbic acid content (from 237.01 to 334.42 mg·100 g^−1^ F.W.) in contrast to others where it decreased. Differences in the vitamin C content of fresh vegetables may be due to a number of different factors, such as cultivar, crop maturity, climatic and agrotechnical conditions, light intensity or harvest time, among others (Becerra-Moreno et al.) [74]. Vitamin C is a potent scavenger and also reducing agent of reactive species in the biological system. Ascorbic acid is included in the first line of defence of antioxidants, protecting the proteins and lipid membrane from damage (Chaudhary et al.) [75]. Due to its water-soluble nature, vitamin C can work both outside and inside the cells and can neutralize the free reactive species to avoid any damage [75, 76].

Technological treatments, such as blanching, boiling and, earlier, pre-treatment (washing, peeling, grinding), among others, can cause significant losses of antioxidant compounds, especially vitamin C. In addition, it is an easily water-soluble vitamin, hence processes carried out in an aqueous environment at elevated temperatures cause large losses of it [46, 77]. Studies by Kapusta-Duch et al. [46] and Sikora and Bodziarczyk [54] showed that the vitamin C content of kale decreased by 79 and 89%, respectively, as a result of conventional boiling, which coincides with the results obtained in this work. The magnitude of losses depends, among other things, on the temperature used and its duration, on the degree of fineness of the product, on the ratio of vegetable weight to the amount of water, and on the method of hydrothermal treatment (traditional cooking, microwave cooking, etc.) [14, 78].

### Carotenoids

Carotenoids are among the most abundant naturally occurring pigments found in plants and plant foods. They are essential for photoprotection and photosynthesis. Kale is an excellent source of dietary carotenoids and typically has the highest concentrations of lutein and zeaxanthin among other green leafy vegetables [79, 80]. Similar results to those obtained in this study were obtained by Korus [81] and Ljubej et al. [82].

The qualitative and quantitative composition of carotenoids in plants is known to vary with multiple factors such as, cultivar or variety, climate, farming practice, maturity at harvest, post-harvest processing and storage conditions [80].

Boiling can lead to the loss of carotenoids as a result of their degradation by heat and water action. Boiling time and temperature are important. Shorter boiling times at lower temperatures can help retain more carotenoids. Steaming is one of the more gentle processes that can minimise the loss of carotenoids. Sometimes food processing can release carotenoids from plant cells. It is worth remembering that some types of carotenoids are more stable than others, and their behaviour during boiling processes may vary.

Boiling was reported to be the most destructive form of heat treatment, and steaming the least. Due to the matrix of plant tissues and the destruction of the integrity of cell walls and membranes, carotenoid extractability is often improved after exposure to certain heat treatments, which may account for the increased concentrations of carotenoids reported, particularly after steaming. Bioavailability is also often increased due to this [79, 80]. In some cases, heat treatments may cause an increase in carotenoids through the release of these components from the carotenoid-protein complexes and changes occurring in proportions of soluble to insoluble components of vegetables [83].

### Polyphenols

Boiling kale can affect the polyphenols content. Longer and more intensive boiling processes can lead to the loss of some polyphenols, especially water-soluble ones. However, shorter boiling processes, such as blanching or steaming, can help retain more of the polyphenols in kale.

Phenolic compounds are not nutrients but, the dietary intake provides health-protective effects, antibacterial, anti-inflammatory, anti-asthmatic, anti-cancer, anti-aging, neuroprotective and anti-mutagenic effects [84]. Kale is reported for 201.67–1167 mg/100 g of total polyphenols content [54, 55, 85–88]. In this study, the average results obtained for the raw vegetables fall within this wide range. The main phenolic compounds found in kale are hydroxycinnamic acids and flavonoid glycosides, including quercetin, kaempferol, derivatives of caffeic, ferulic and sinapic acids [12, 89].

In this study, thermal processes resulted in a systematic reduction in the total polyphenols content of the analysed kale of both cultivars. In the studies of Kapusta-Duch et al. [46], boiling of kale caused a 36.9% fall in total polyphenols, which was lower than the losses found by Sikora and Bodziarczyk [54] (56%) and those reported by Sultana et al. [90] (43%). Thermal processing significantly reduced the phenolic content of kale also in study of Lafarga et al. [91].

### ABTS^●+^

The antioxidant activity of the compounds contained in raw, blanched and cooked kale was determined on the basis of the free radical quenching capacity of ABTS^●+^ by the methanolic extracts of the test vegetable and expressed as TEAC (Trolox Equivalent Antioxidant Capacity). The average antioxidant activity of the control kale before culinary-technological processing was 17 μmols of Trolox/ g fresh weight of the analysed vegetable, which coincides with the results reported by other authors, i.e. Sikora and Bodziarczyk [54] 22.06–47.38, Korus [72] 17.6, Sikora et al. [92] 36.2 or Kapusta-Duch [67] 20.5 μmols of Trolox/ g fresh weight. Discrepancies in the aforementioned values may be due to a number of factors, including: the content of individual antioxidants, their interactions with each other, the cultivar and species of the plant, growing conditions, harvesting date, fertilisers used, and the method of sampling. The type of extraction performed is also an important aspect that can affect the differences in results. The antioxidant activity of a food is related to the content of substances in it that have the ability to react with free radicals, primarily vitamin C, E, β-carotene and polyphenols [75]. The calculation results obtained may also vary, depending on the methodology for the determination of a given quantity (ABTS, DPPH, FRAP, ORAC, etc.), as well as the measuring equipment used, or the other way of expressing the antioxidant potential (percentage of free radical reduction qualitative, Trolox equivalent quantitative).

In this study, for the control kale cultivars ‘Oldenbor F_1_’ and ‘Redbor F_1_’, the processes of steaming, blanching and traditional boiling in water resulted in a reduction in antioxidant activity of 54%, 48% and 85%, respectively, compared to kale before technological treatment (the average percentage was calculated from two cultivars). Sikora and Bodziarczyk [54] observed a 38% reduction in the antioxidant activity of kale under the influence of the boiling process. A much smaller loss is reported by the team of Kapusta-Duch et al. [46], by only 0.5%. In the study of Murador et al. [93], according to the ABTS^●+^ assay, steaming resulted in notably significant increases in antioxidant activity in kale (186.9%; p < 0.0001). This effect was also confirmed by the ORAC assay, and the steamed sample showed an increase of 50.7% (p < 0.0001) compared with the raw sample. On the other hand, authors reported that the conventional boiling showed a significant decrease in antioxidant activity in kale (43.3%; p < 0.0001). Other authors also observed reductions of antioxidant activity of the processed *Brassica* vegetables [94–96]. Technological processes to which *Brassica* vegetable are usually subjected lead to changes in the antioxidant activity. Boiling of vegetables causes a large decrease in antioxidant activity due to the loss of vitamin C and polyphenols, which dissolve in water. These losses are the greatest in the case of leafy or highly fragmented vegetables like kale [91].

## Conclusions

Iodine applied in the form of KIO_3_, 8-OH-7-I-5QSA, and 5-Cl-7-I-8-Q was taken up by the roots and transported to the kale leaves. When plants were grown in a hydroponic cultivation, the application of 8-OH-7-I-5QSA and 5-Cl-7-I-8-Q showed significantly better iodine biofortification efficiency, % RDA-I, and iodine uptake by leaves compared to the control. High iodine biofortification efficiency, % RDA-I, and iodine uptake by leaves were also shown by KIO_3_, compared to the control and iodoquinolines. 8-OH-7-I-5QSA, iodine biofortification efficiency, % RDA-I, and iodine uptake by leaves were at a higher level than KIO_3_, but in contrast, 5-Cl-7-I-8-Q was lower than KIO_3_.

The use of 8-OH-7-I-5QSA and 5-Cl-7-I-8-Q for plant biofortification is economically justified. However, the implementation of iodoquinolines into agricultural practice still requires further research to determine the effects of plants enriched with these compounds on consumer organisms. The bioavailability of this trace element should be verified by in vivo studies.

There was no significant negative effect of the applied iodine compounds on the basic composition of kale leaves of both ‘Oldenbor F_1_’ and ‘Redbor F_1_’ cultivars. It has been shown that biofortification can increase the content of polyphenols, and carotenoids and improve the antioxidant activity of the plant.

This study clearly shows that the nutrients and bioactive components of kale of both cultivars (‘Oldenbor F_1_’, ‘Redbor F_1_’), are significantly affected by losses during conventional steaming, blanching and boiling processes. The thermal processes have been shown to significantly affect iodine content, chemical composition, and vitamin C, polyphenols, ABTS^●+^ and carotenoids. We found a significant decrease in total polyphenols, ABTS^●+^ and ascorbic acid content in kale of both cultivars (‘Oldenbor F_1_’, ‘Redbor F_1_’) biofortified with iodine (KIO_3_, 8-hydroxy-7-iodo-5-quinolinesulfonic acid and 5-chloro-7-iodo-8-quinolinol) under steaming, blanching and boiling processes compared to raw biofortified kale.

We recommend consuming non-biofortified and biofortified kale in raw form for salads, as it best retains its nutritional values, including iodine, under these conditions. With a high intake of kale, due to its wide range of health-promoting properties, we can reduce the occurrence of NCDs, taking into account the most optimal cooking technique.

### Patents

The method of biofortification of vegetables in cultivated using traditional, pot experiment, hydroponic method with the using 8-Hydroxy-7-iodo-5-quinolinesulfonic acid and of 5-Chloro-7-iodo-8-quinolinol for biofortification of vegetables with iodine was patented. The method of enriching plants with iodoquinolines protected by patent law by the Polish Patent Office. Patent applications No. P.443218 for 8-OH-7-I-5QSA and P.443220 for 5-Cl-7-I-8-Q (21 December 2022).

## Supporting information

S1 Fig **A.** Iodine μg∙kg^-1^ F.W. of curly kale leaves ‘Oldenbor F_1_’ and ‘Redbor F_1_’ in fresh weight before and after heat treatment; means followed by different letters for treatments, differ significantly at *p* < 0.05 (Duncan’s post-hoc test); bars indicate standard error (n = 4). Homogeneous groups refer to a three-factor analysis of variance: factor No. 1 culinary treatment: raw, steaming, blanching, boiling x factor No. 2 type of enrichment: control, KIO_3_, 8-OH-7-I-5QSA, 5-Cl-7-I-8-Q x factor No. 3 kale cultivar: ’Oldenbor F_1_’ and ’Redbor F_1_’. **B.** Iodine content (mg∙L^-1^) of the water after boiling; means followed by different letters for treatments, differ significantly at *p* < 0.05 (Duncan’s post-hoc test); bars indicate standard error (n = 4). Homogeneous groups refer to two-factor analysis of variance: factor No. 1 type of enrichment: control, KIO_3_, 8-OH-7-I-5QSA, 5-Cl-7-I-8-Q, x factor No. 2 kale cultivar: ’Oldenbor F_1_’ and ’Redbor F_1_’.
(TIF)

S2 Fig Chemical composition of leaves curly kale ‘Oldenbor F_1_’ and ‘Redbor F_1_’ in fresh weight before and after heat treatment; means followed by different letters for treatments, differ significantly at *p* < 0.05 (Duncan’s post-hoc test); bars indicate standard error (n = 3). **A.** Dry matter g∙100 g^-1^ F.W.; **B.** Ash g∙100 g^-1^ F.W.; **C.** Protein g∙100 g^-1^ F.W.; **D.** Fat g∙100 g^-1^ F.W.; **E.** Total Carbohydrate g∙100 g^-1^ F.W.; **F.** Dietary fiber g∙100 g^-1^ F.W. Homogeneous groups refer to a three-factor analysis of variance: factor No. 1 culinary treatment: raw, steaming, blanching, boiling x factor No. 2 type of enrichment: control, KIO_3_, 8-OH-7-I-5QSA, 5-Cl-7-I-8-Q x factor No. 3 kale cultivar: ’Oldenbor F_1_’ and ’Redbor F_1_’.
(TIF)

S3 Fig The antioxidant activity (**B**) and content of total polyphenols (**A**), total carotenoids (**D**), and ascorbic acid (**C**) in leaves curly kale ‘Oldenbor F_1_’ and ‘Redbor F_1_’ before and after heat treatment; means followed by different letters for treatments, differ significantly at *p* < 0.05 (Duncan’s post-hoc test); bars indicate standard error (n = 3). Homogeneous groups refer to a three-factor analysis of variance: factor No. 1 culinary treatment: raw, steaming, blanching, boiling x factor No. 2 type of enrichment: control, KIO_3_, 8-OH-7-I-5QSA, 5-Cl-7-I-8-Q x factor No. 3 kale cultivar: ’Oldenbor F_1_’ and ’Redbor F_1_’.
(TIF)

S4 Fig Percentage coverage of Recommended Daily Allowance for iodine (% RDA-I) and hazard quotient (HQ) for intake of iodine through consumption of 100 g and 50 g portions of leaves curly kale ‘Oldenbor F_1_’ and ‘Redbor F_1_’ before and after heat treatment in individual by adults 70 kg body weight; means followed by different letters for treatments, differ significantly at *p* < 0.05 (Duncan’s post-hoc test); bars indicate standard error (n = 4). **A.** Daily Intake of I with 50 g of kale (μg I·day^−1^); **B.** Daily Intake of I with 100 g of kale (μg I·day^−1^); **C.** % RDA I (in 50 g portion of kale); **D.** % RDA I (in 100 g portion of kale); **E.** HQ for 50 g portion of kale; **F.** HQ for 100 g portion of kale. Homogeneous groups refer to a three-factor analysis of variance: factor No. 1 culinary treatment: raw, steaming, blanching, boiling x factor No. 2 type of enrichment: control, KIO_3_, 8-OH-7-I-5QSA, 5-Cl-7-I-8-Q x factor No. 3 kale cultivar: ’Oldenbor F_1_’ and ’Redbor F_1_’.
(TIF)

S1 Data (PDF)

S2 Data (PDF)

S3 Data (JPG)

S4 Data (JPG)

S1 File (XLSX)
