# Supplementary figures and images for: Iodine enriched kale (Brassica oleracea var. sabellica L.)—The influence of heat treatments on its iodine content, basic composition and antioxidative properties
Source: PLoS One. 2024 Jun 27;19(6):e0304005. doi: 10.1371/journal.pone.0304005 (PMC11210757; doi:10.1371/journal.pone.0304005)

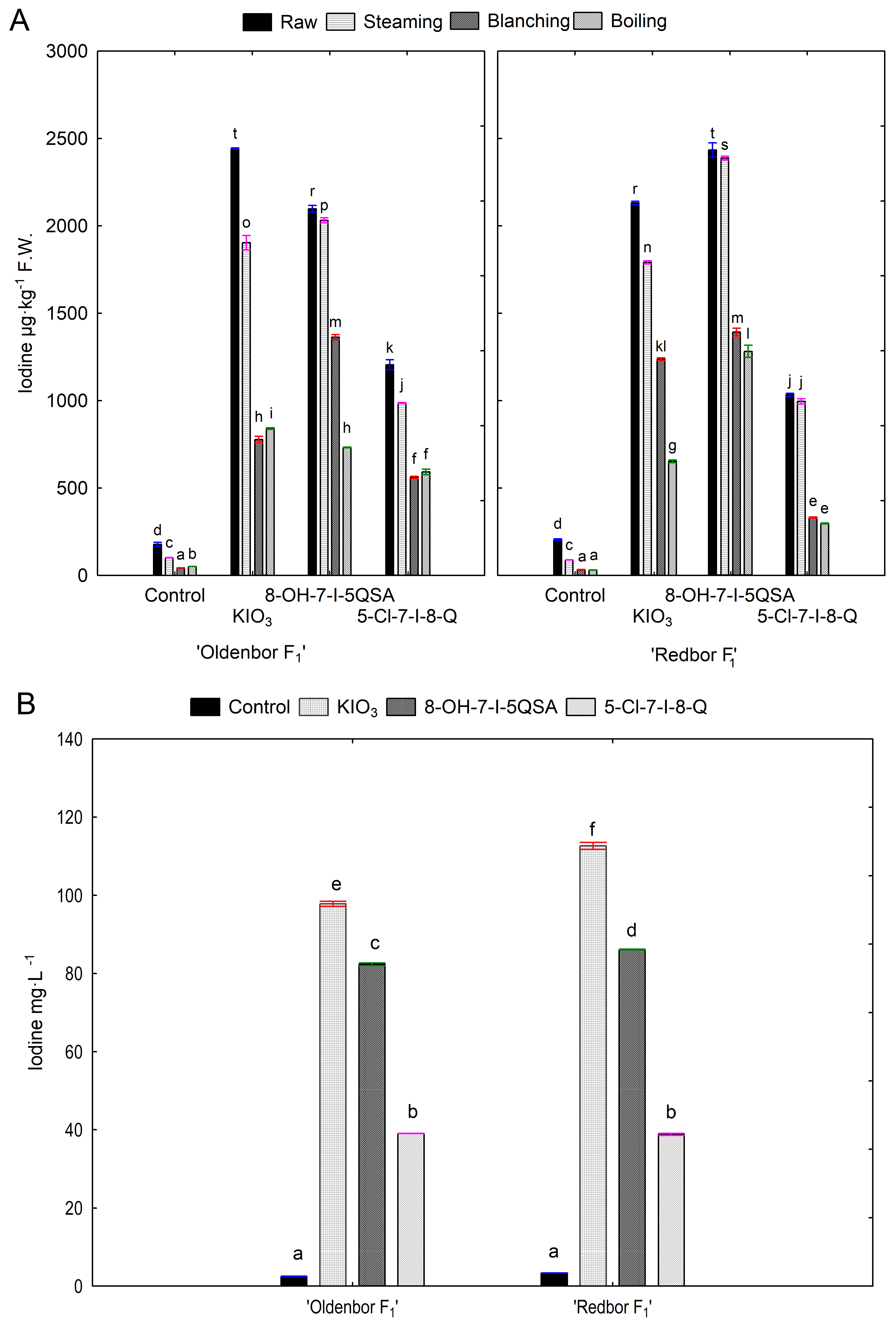

Supplement: S1 Fig — A. Iodine μg∙kg-1 F.W. of curly kale leaves ‘Oldenbor F1’ and ‘Redbor F1’ in fresh weight before and after heat treatment; means followed by different letters for treatments, differ significantly at p < 0.05 (Duncan’s post-hoc test); bars indicate standard error (n = 4). Homogeneous groups refer to a three-factor analysis of variance: factor No. 1 culinary treatment: raw, steaming, blanching, boiling x factor No. 2 type of enrichment: control, KIO3, 8-OH-7-I-5QSA, 5-Cl-7-I-8-Q x factor No. 3 kale cultivar: ’Oldenbor F1’ and ’Redbor F1’. B. Iodine content (mg∙L-1) of the water after boiling; means followed by different letters for treatments, differ significantly at p < 0.05 (Duncan’s post-hoc test); bars indicate standard error (n = 4). Homogeneous groups refer to two-factor analysis of variance: factor No. 1 type of enrichment: control, KIO3, 8-OH-7-I-5QSA, 5-Cl-7-I-8-Q, x factor No. 2 kale cultivar: ’Oldenbor F1’ and ’Redbor F1’. (TIF) [file pone.0304005.s001.tif]

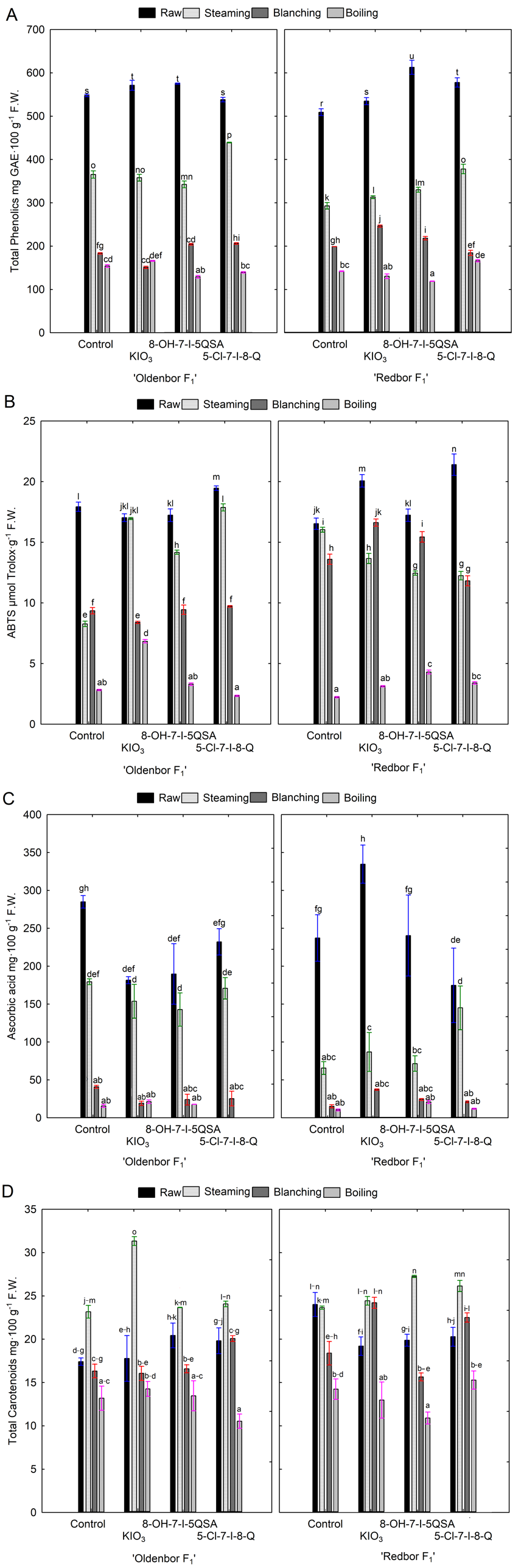

Supplement: S3 Fig — The antioxidant activity (B) and content of total polyphenols (A), total carotenoids (D), and ascorbic acid (C) in leaves curly kale ‘Oldenbor F1’ and ‘Redbor F1’ before and after heat treatment; means followed by different letters for treatments, differ significantly at p < 0.05 (Duncan’s post-hoc test); bars indicate standard error (n = 3). Homogeneous groups refer to a three-factor analysis of variance: factor No. 1 culinary treatment: raw, steaming, blanching, boiling x factor No. 2 type of enrichment: control, KIO3, 8-OH-7-I-5QSA, 5-Cl-7-I-8-Q x factor No. 3 kale cultivar: ’Oldenbor F1’ and ’Redbor F1’. (TIF) [file pone.0304005.s003.tif]

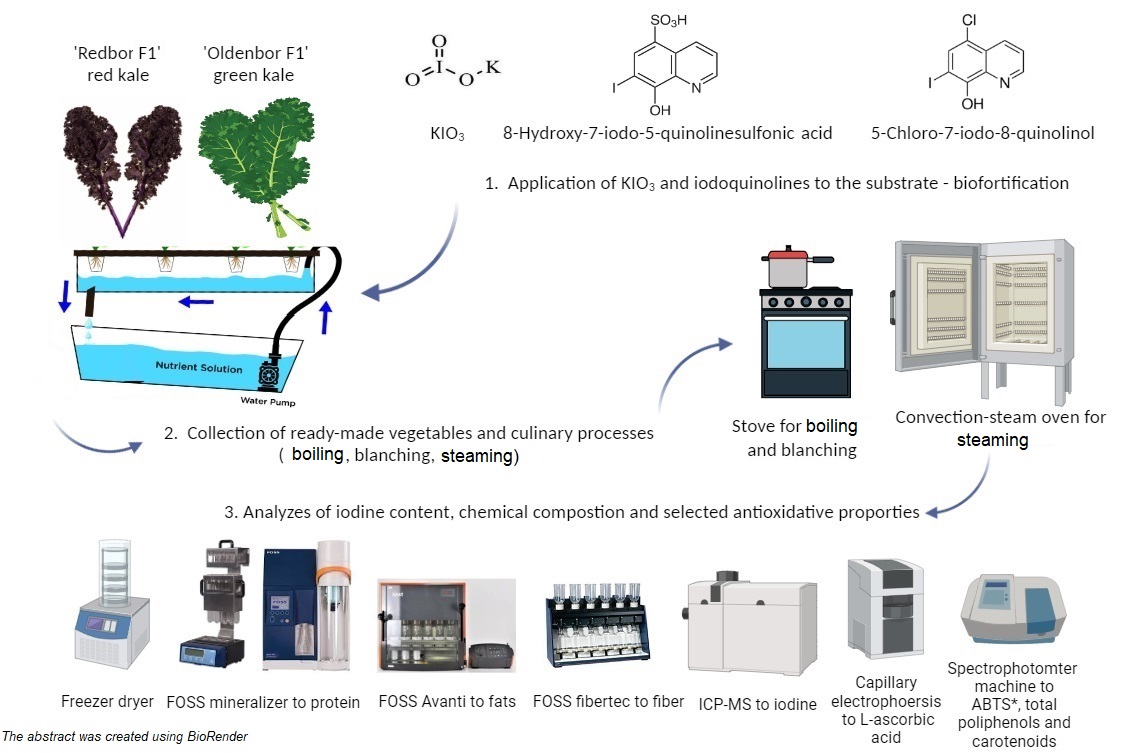

Supplement: S3 Data — (JPG) [file pone.0304005.s007.jpg]
