# Supplementary material for: Iodine enriched kale (Brassica oleracea var. sabellica L.)—The influence of heat treatments on its iodine content, basic composition and antioxidative properties
Source: PLoS One. 2024 Jun 27;19(6):e0304005. doi: 10.1371/journal.pone.0304005 (PMC11210757; doi:10.1371/journal.pone.0304005)
Supplement: S1 Data — (PDF) [file pone.0304005.s005.pdf]

Supplementary Material

Iodine enriched kale (*Brassica oleracea* var. *sabellica* L.) the influence of heat treatments on its iodine content, basic composition and antioxidative properties

Krzemińska Joanna [1*], Kapusta-Duch Joanna [1], Smoleń Sylwester [2], Kowalska Iwona [2], Słupski Jacek [3], Skoczeń-Słupska Radosława [3], Krawczyk Katarzyna [1], Waśniowska Justyna [1], and Koronowicz Aneta [1*]

[1] Department of Human Nutrition and Dietetics, Faculty of Food Technology, University of Agriculture in Krakow, Balicka 122, 30-149 Krakow, Poland;

[2] Department of Plant Biology and Biotechnology, Faculty of Biotechnology and Horticulture, University of Agriculture in Krakow, Al. 29 Listopada 54, 31-425 Krakow, Poland;

[3] Department of Plant Product Technology and Nutrition Hygiene, Faculty of Food Technology, University of Agriculture in Krakow, Balicka 122, 30-149 Krakow, Poland;

[*]Correspondence: aneta.koronowicz@urk.edu.pl; Tel.: +48-12-662-48-31, joanna.krzemińska@urk.edu.pl; Tel.: +48-12-662-48-19

List of tables in the supporting information file

1. **Figure S1. A.** Iodine μg·kg^{-1} F.W. of curly kale leaves 'Oldenbor F_1' and 'Redbor F_1' in fresh weight before and after heat treatment; means followed by different letters for treatments, differ significantly at $p < 0.05$ (Duncan's post-hoc test); bars indicate standard error (n = 4). Homogeneous groups refer to a three-factor analysis of variance: factor No. 1 culinary treatment: raw, steaming, blanching, boiling x factor No. 2 type of enrichment: control, KIO_3, 8-OH-7-I-5QSA, 5-Cl-7-I-8-Q x factor No. 3 kale cultivar: 'Oldenbor F_1' and 'Redbor F_1'. **B.** Iodine content (mg·L^{-1}) of the water after boiling; means followed by different letters for treatments, differ significantly at $p < 0.05$ (Duncan's post-hoc test); bars indicate standard error (n = 4). Homogeneous groups refer to two-factor analysis of variance: factor No. 1 type of enrichment: control, KIO_3, 8-OH-7-I-5QSA, 5-Cl-7-I-8-Q, x factor No. 2 kale cultivar: 'Oldenbor F_1' and 'Redbor F_1'.

2. **Figure S2.** Chemical composition of leaves curly kale 'Oldenbor F_1' and 'Redbor F_1' in fresh weight before and after heat treatment; means followed by different letters for treatments, differ significantly at $p < 0.05$ (Duncan's post-hoc test); bars indicate standard error (n = 3). **A.** Dry matter g·100 g^{-1} F.W.; **B.** Ash g·100 g^{-1} F.W.; **C.** Protein g·100 g^{-1} F.W.; **D.** Fat g·100 g^{-1} F.W.; **E.** Total Carbohydrate g·100 g^{-1} F.W.; **F.** Dietary fiber g·100 g^{-1} F.W. Homogeneous groups refer to a three-factor analysis of variance: factor No. 1 culinary treatment: raw, steaming, blanching, boiling x factor No. 2 type of enrichment: control, KIO_3, 8-OH-7-I-5QSA, 5-Cl-7-I-8-Q x factor No. 3 kale cultivar: 'Oldenbor F_1' and 'Redbor F_1'.

3. **Figure S3.** The antioxidant activity (**B**) and content of total polyphenols (**A**), total carotenoids (**D**), and ascorbic acid (**C**) in leaves curly kale 'Oldenbor F_1' and 'Redbor F_1' before and after heat

treatment; means followed by different letters for treatments, differ significantly at $p < 0.05$ (Duncan's post-hoc test); bars indicate standard error (n = 3). Homogeneous groups refer to a three-factor analysis of variance: factor No. 1 culinary treatment: raw, steaming, blanching, boiling x factor No. 2 type of enrichment: control, KIO_3, 8-OH-7-I-5QSA, 5-Cl-7-I-8-Q x (3) kale cultivar: 'Oldenbor F_1' and 'Redbor F_1'.

4. **Figure S4.** Percentage coverage of Recommended Daily Allowance for iodine (% RDA-I) and hazard quotient (HQ) for intake of iodine through consumption of 100 g and 50 g portions of leaves curly kale 'Oldenbor F_1' and 'Redbor F_1' before and after heat treatment in individual by adults 70 kg body weight; means followed by different letters for treatments, differ significantly at $p < 0.05$ (Duncan's post-hoc test); bars indicate standard error (n = 4). **A.** Daily Intake of I with 50 g of kale ($\mu g\ I \cdot day^{-1}$); **B.** Daily Intake of I with 100 g of kale ($\mu g\ I \cdot day^{-1}$); **C.** % RDA I (in 50 g portion of kale); **D.** % RDA I (in 100 g portion of kale); **E.** HQ for 50 g portion of kale; **F.** HQ for 100 g portion of kale. Homogeneous groups refer to a three-factor analysis of variance: factor No. 1 culinary treatment: raw, steaming, blanching, boiling x factor No. 2 type of enrichment: control, KIO_3, 8-OH-7-I-5QSA, 5-Cl-7-I-8-Q x factor No. 3 kale cultivar: 'Oldenbor F_1' and 'Redbor F_1'.

Figure S1. A. Iodine µg·kg^{-1} F.W. of curly kale leaves 'Oldenbor F$_1$' and 'Redbor F$_1$' in fresh weight before and after heat treatment; means followed by different letters for treatments, differ significantly at $p < 0.05$ (Duncan's post-hoc test); bars indicate standard error (n = 4). Homogeneous groups refer to a three-factor analysis of variance: factor No. 1 culinary treatment: raw, steaming, blanching, boiling x factor No. 2 type of enrichment: control, KIO3, 8-OH-7-I-5QSA, 5-Cl-7-I-8-Q x factor No. 3 kale cultivar: 'Oldenbor F$_1$' and 'Redbor F$_1$'. **B.** Iodine content (mg·L^{-1}) of the water after boiling; means followed by different letters for treatments, differ significantly at $p < 0.05$ (Duncan's post-hoc test); bars indicate standard error (n = 4). Homogeneous groups refer to two-factor analysis of variance: factor No. 1 type of enrichment: control, KIO3, 8-OH-7-I-5QSA, 5-Cl-7-I-8-Q, x factor No. 2 kale cultivar: 'Oldenbor F$_1$' and 'Redbor F$_1$'.

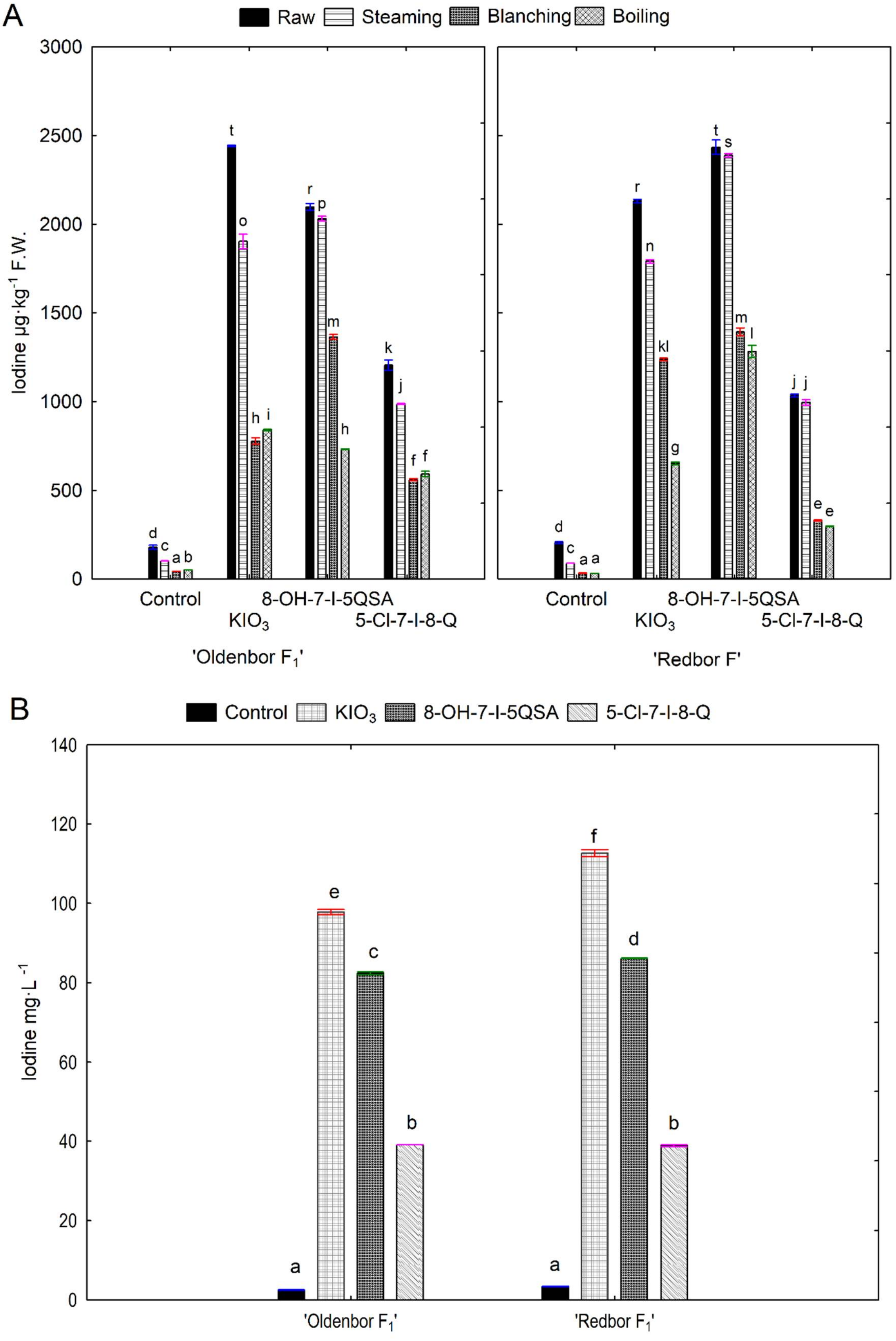

Table 1. Content of Iodine µg·kg^{-1} F.W. of curly kale leaves 'Oldenbor F_1' and 'Redbor F_1' with or without biofortification before and after heat treatment.

Culinary treatment	Enrichment	Cultivar	Iodine µg·kg^{-1} F.W.
Raw	Control	'Oldenbor F_1'	178.49 ± 23.68 ^{d}
		'Redbor F_1'	204.49 ± 10.84 ^{d}
	KIO_3	'Oldenbor F_1'	2442.59 ± 7.22 ^{t}
		'Redbor F_1'	2130.97 ± 20.73 ^{r}
	8-OH-7-I-5QSA	'Oldenbor F_1'	2097.98 ± 37.64 ^{r}
		'Redbor F_1'	2434.70 ± 82.14 ^{t}
	5-Cl-7-I-8-Q	'Oldenbor F_1'	1206.00 ± 57.45 ^{k}
		'Redbor F_1'	1034.00 ± 18.18 ^{j}
Steaming	Control	'Oldenbor F_1'	103.24 ± 3.35 ^{c}
		'Redbor F_1'	89.11 ± 1.72 ^{c}
	KIO_3	'Oldenbor F_1'	1903.96 ± 82.48 ^{o}
		'Redbor F_1'	1793.85 ± 18.19 ^{n}
	8-OH-7-I-5QSA	'Oldenbor F_1'	2031.58 ± 29.61 ^{p}
		'Redbor F_1'	2388.01 ± 20.90 ^{s}
	5-Cl-7-I-8-Q	'Oldenbor F_1'	987.34 ± 6.59 ^{j}
		'Redbor F_1'	998.36 ± 31.57 ^{j}
Blanching	Control	'Oldenbor F_1'	43.41 ± 1.05 ^{a}
		'Redbor F_1'	33.38 ± 1.31 ^{a}
	KIO_3	'Oldenbor F_1'	779.32 ± 35.89 ^{h}
		'Redbor F_1'	1242.94 ± 15.59 ^{kl}
	8-OH-7-I-5QSA	'Oldenbor F_1'	1377.32 ± 26.32 ^{m}
		'Redbor F_1'	1397.83 ± 45.34 ^{m}
	5-Cl-7-I-8-Q	'Oldenbor F_1'	562.23 ± 13.20 ^{f}
		'Redbor F_1'	331.62 ± 8.16 ^{e}
Boiling	Control	'Oldenbor F_1'	51.90 ± 0.78 ^{b}
		'Redbor F_1'	30.81 ± 1.07 ^{a}
	KIO_3	'Oldenbor F_1'	842.13 ± 8.56 ^{i}
		'Redbor F_1'	658.75 ± 16.05 ^{g}
	8-OH-7-I-5QSA	'Oldenbor F_1'	745.99 ± 3.61 ^{h}
		'Redbor F_1'	1287.78 ± 70.53 ^{l}
	5-Cl-7-I-8-Q	'Oldenbor F_1'	593.71 ± 30.96 ^{f}
		'Redbor F_1'	299.29 ± 4.24 ^{e}

Results are shown as mean ± standard error (SE); n = 4; homogeneous groups refer to a three-factor analysis of variance: factor No. 1 culinary treatment: raw, steaming, blanching, boiling x factor No. 2 type of enrichment: control, KIO_3, 8-OH-7-I-5QSA, 5-Cl-7-I-8-Q x factor No. 3 kale cultivar: 'Oldenbor F_1' and 'Redbor F_1'; means followed by the same letter are not significantly different $p < 0.05$ (Duncan's post-hoc test).

Table 2. Iodine content (mg·L^{-1}) of the water after boiling the of curly kale leaves of both cultivars 'Oldenbor F_1' and 'Redbor F_1' with or without biofortification.

Enrichment	Cultivar	Iodine mg·L^{-1}
Control	'Oldenbor F_1'	2.48±0.07 ^{a}
	'Redbor F_1'	3.37±0.04 ^{a}
KIO_3	'Oldenbor F_1'	97.84±0.9 ^{e}
	'Redbor F_1'	112.63±1.24 ^{f}
8-OH-7-I-5QSA	'Oldenbor F_1'	82.33±0.5 ^{c}
	'Redbor F_1'	86.09±0.14 ^{d}
5-Cl-7-I-8-Q	'Oldenbor F_1'	39.06±0.04 ^{b}
	'Redbor F_1'	38.83±0.4 ^{b}

Results are shown as mean ± standard error (SE); n = 4; homogeneous groups refer to two-factor analysis of variance: factor No. 1 type of enrichment: control, KIO_3, 8-OH-7-I-5QSA, 5-Cl-7-I-8-Q, x factor No. 2 kale cultivar 'Oldenbor F_1' and 'Redbor F_1'; means followed by the same letter are not significantly different $p < 0.05$ (Duncan's post-hoc test).

Figure S2. Chemical composition of leaves curly kale ‘Oldenbor F_1’ and ‘Redbor F_1’ in fresh weight before and after heat treatment; means followed by different letters for treatments, differ significantly at $p < 0.05$ (Duncan’s post-hoc test); bars indicate standard error (n = 3). **A.** Dry matter g·100 g^{-1} F.W.; **B.** Ash g·100 g^{-1} F.W.; **C.** Protein g·100 g^{-1} F.W.; **D.** Fat g·100 g^{-1} F.W.; **E.** Total Carbohydrate g·100 g^{-1} F.W.; **F.** Dietary fiber g·100 g^{-1} F.W. Homogeneous groups refer to a three-factor analysis of variance: factor No. 1 culinary treatment: raw, steaming, blanching, boiling x factor No. 2 type of enrichment: control, KIO_3, 8-OH-7-I-5QSA, 5-Cl-7-I-8-Q x factor No. 3 kale cultivar: 'Oldenbor F_1' and 'Redbor F_1'.

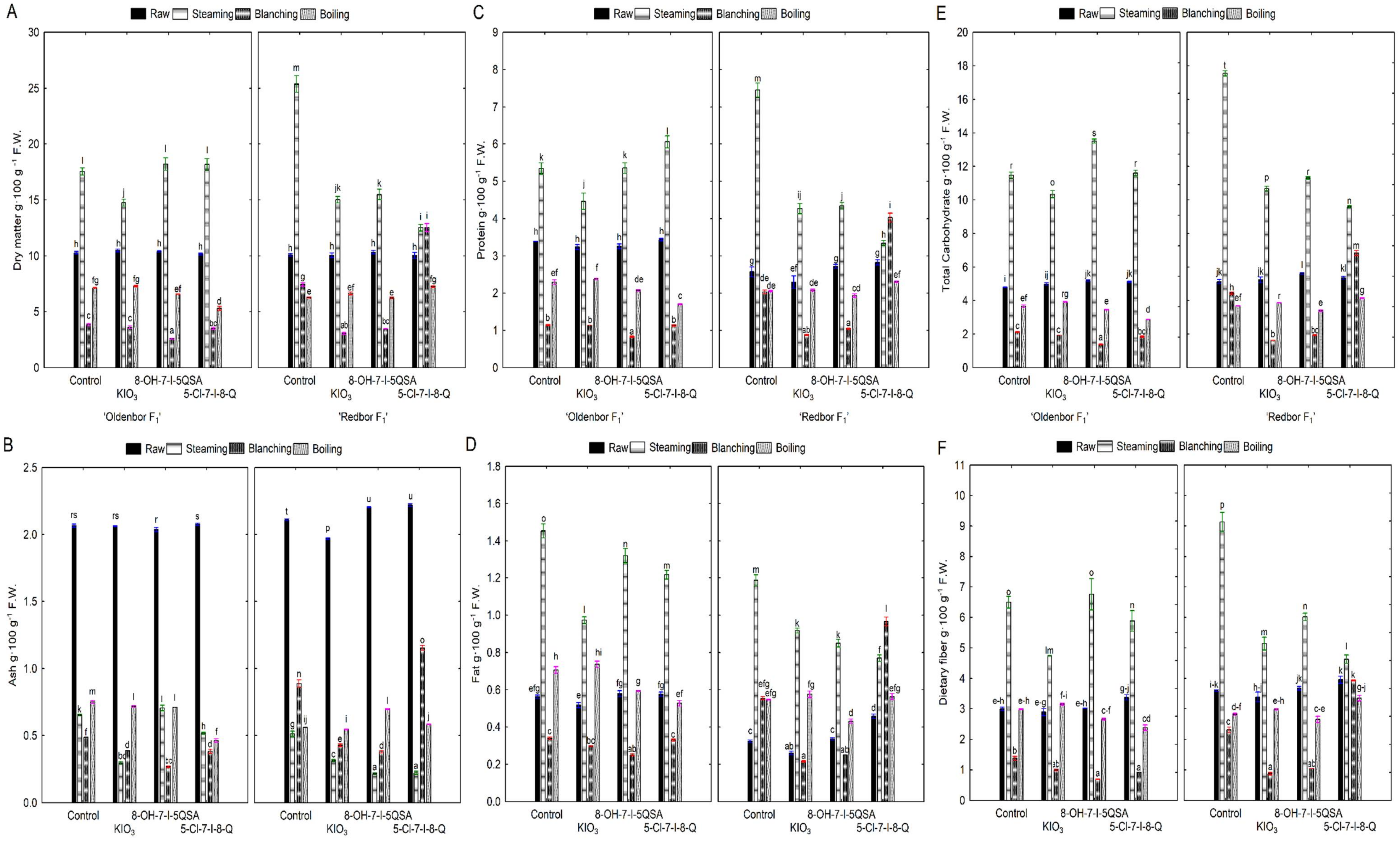

Table 3. Chemical composition of kale 'Oldenbor F_1' and 'Redbor F_1' in fresh tissue before and after heat treatment.

Culinary treatment	Enrichment	Cultivar	Dry matter g·100 g^{-1} F.W.	Ash g·100 g^{-1} F.W.	Protein g·100 g^{-1} F.W.	Fat g·100 g^{-1} F.W.	Total Carbohydrate g·100 g^{-1} F.W.	Dietary fiber g·100 g^{-1} F.W.
Raw	Control	'Oldenbor F_1'	10.30 ± 0.22 ^{h}	2.07 ± 0.02 ^{rs}	3.39 ± 0.01 ^{h}	0.56 ± 0.02 ^{efg}	4.78 ± 0.04 ^{i}	3.00 ± 0.08 ^{efgh}
		'Redbor F_1'	10.10 ± 0.23 ^{h}	2.11 ± 0.01 ^{t}	2.58 ± 0.23 ^{g}	0.32 ± 0.01 ^{c}	5.13 ± 0.22 ^{jk}	3.60 ± 0.04 ^{ijk}
	KIO_3	'Oldenbor F_1'	10.50 ± 0.24 ^{h}	2.06 ± 0.01 ^{rs}	3.24 ± 0.11 ^{h}	0.52 ± 0.02 ^{e}	4.99 ± 0.13 ^{ij}	2.89 ± 0.17 ^{efg}
		'Redbor F_1'	10.08 ± 0.39 ^{h}	1.97 ± 0.01 ^{p}	2.30 ± 0.30 ^{ef}	0.26 ± 0.02 ^{ab}	5.24 ± 0.31 ^{jk}	3.40 ± 0.23 ^{hij}
	8-OH-7-I-5QSA	'Oldenbor F_1'	10.40 ± 0.14 ^{h}	2.04 ± 0.03 ^{r}	3.26 ± 0.11 ^{h}	0.58 ± 0.03 ^{fg}	5.18 ± 0.08 ^{jk}	3.01 ± 0.01 ^{efgh}
		'Redbor F_1'	10.35 ± 0.30 ^{h}	2.20 ± 0.01 ^{u}	2.72 ± 0.09 ^{g}	0.34 ± 0.01 ^{c}	5.63 ± 0.11 ^{l}	3.68 ± 0.08 ^{jk}
	5-Cl-7-I-8-Q	'Oldenbor F_1'	10.18 ± 0.15 ^{h}	2.08 ± 0.02 ^{s}	3.44 ± 0.06 ^{h}	0.58 ± 0.02 ^{fg}	5.11 ± 0.06 ^{jk}	3.38 ± 0.13 ^{ghij}
		'Redbor F_1'	10.05 ± 0.54 ^{h}	2.22 ± 0.02 ^{u}	2.83 ± 0.12 ^{g}	0.46 ± 0.02 ^{d}	5.36 ± 0.15 ^{kl}	3.96 ± 0.17 ^{k}
Steaming	Control	'Oldenbor F_1'	17.54 ± 0.55 ^{l}	0.65 ± 0.01 ^{k}	5.35 ± 0.26 ^{k}	1.45 ± 0.07 ^{o}	11.47 ± 0.31 ^{r}	6.50 ± 0.28 ^{o}
		'Redbor F_1'	25.38 ± 1.28 ^{m}	0.51 ± 0.04 ^{g}	7.45 ± 0.33 ^{m}	1.19 ± 0.05 ^{m}	17.56 ± 0.27 ^{t}	9.14 ± 0.45 ^{p}
	KIO_3	'Oldenbor F_1'	14.75 ± 0.54 ^{j}	0.30 ± 0.02 $^{b.c}$	4.47 ± 0.38 ^{j}	0.97 ± 0.03 ^{l}	10.34 ± 0.37 ^{o}	4.76 ± 0.01 ^{lm}
		'Redbor F_1'	15.03 ± 0.49 ^{jk}	0.31 ± 0.02 ^{c}	4.27 ± 0.23 ^{ij}	0.92 ± 0.02 ^{k}	10.68 ± 0.25 ^{p}	5.14 ± 0.30 ^{m}
	8-OH-7-I-5QSA	'Oldenbor F_1'	18.21 ± 0.96 ^{l}	0.71 ± 0.04 ^{l}	5.36 ± 0.24 ^{k}	1.32 ± 0.07 ^{n}	13.50 ± 0.22 ^{s}	6.76 ± 0.72 ^{o}
		'Redbor F_1'	15.50 ± 0.81 ^{k}	0.22 ± 0.01 ^{a}	4.34 ± 0.15 ^{j}	0.85 ± 0.03 ^{k}	11.32 ± 0.13 ^{r}	6.02 ± 0.18 ^{n}
	5-Cl-7-I-8-Q	'Oldenbor F_1'	18.19 ± 0.91 ^{l}	0.52 ± 0.02 ^{h}	6.06 ± 0.29 ^{l}	1.22 ± 0.04 ^{m}	11.59 ± 0.31 ^{r}	5.89 ± 0.47 ^{n}
		'Redbor F_1'	12.53 ± 0.50 ^{i}	0.22 ± 0.02 ^{a}	3.34 ± 0.12 ^{h}	0.77 ± 0.03 ^{i}	9.59 ± 0.11 ^{n}	4.63 ± 0.20 ^{l}
Blanching	Control	'Oldenbor F_1'	3.84 ± 0.16 ^{c}	0.49 ± 0.00 ^{f}	1.14 ± 0.03 ^{b}	0.34 ± 0.01 ^{c}	2.14 ± 0.03 ^{c}	1.39 ± 0.09 ^{b}
		'Redbor F_1'	7.44 ± 0.27 ^{g}	0.89 ± 0.05 ^{n}	2.03 ± 0.09 ^{de}	0.55 ± 0.02 ^{efg}	4.46 ± 0.11 ^{h}	2.32 ± 0.13 ^{c}
	KIO_3	'Oldenbor F_1'	3.63 ± 0.19 ^{c}	0.39 ± 0.00 ^{d}	1.14 ± 0.01 ^{b}	0.30 ± 0.01 ^{bc}	1.96 ± 0.01 ^{c}	1.00 ± 0.04 ^{ab}
		'Redbor F_1'	3.05 ± 0.17 ^{ab}	0.43 ± 0.02 ^{e}	0.88 ± 0.03 ^{ab}	0.22 ± 0.01 ^{a}	1.67 ± 0.02 ^{b}	0.89 ± 0.06 ^{a}
	8-OH-7-I-5QSA	'Oldenbor F_1'	2.58 ± 0.10 ^{a}	0.27 ± 0.01 ^{bc}	0.83 ± 0.03 ^{a}	0.25 ± 0.01 ^{ab}	1.39 ± 0.04 ^{a}	0.72 ± 0.01 ^{a}
		'Redbor F_1'	3.48 ± 0.03 ^{bc}	0.38 ± 0.02 ^{d}	1.05 ± 0.03 ^{a}	0.25 ± 0.00 ^{ab}	1.94 ± 0.05 ^{bc}	1.06 ± 0.01 ^{ab}
	5-Cl-7-I-8-Q	'Oldenbor F_1'	3.53 ± 0.16 ^{bc}	0.38 ± 0.03 ^{d}	1.14 ± 0.03 ^{b}	0.33 ± 0.01 ^{c}	1.86 ± 0.07 ^{bc}	0.92 ± 0.00 ^{a}
		'Redbor F_1'	12.54 ± 0.62 ^{i}	1.16 ± 0.03 ^{o}	4.02 ± 0.20 ^{i}	0.97 ± 0.04 ^{l}	6.85 ± 0.26 ^{m}	3.94 ± 0.02 ^{k}
Boiling	Control	'Oldenbor F_1'	7.13 ± 0.08 ^{fg}	0.75 ± 0.02 ^{m}	2.29 ± 0.11 ^{ef}	0.71 ± 0.03 ^{h}	3.66 ± 0.13 ^{ef}	2.99 ± 0.02 ^{efgh}
		'Redbor F_1'	6.26 ± 0.11 ^{e}	0.56 ± 0.00 ^{ij}	2.05 ± 0.03 ^{de}	0.55 ± 0.01 ^{efg}	3.69 ± 0.03 ^{ef}	2.82 ± 0.05 ^{def}
	KIO_3	'Oldenbor F_1'	7.28 ± 0.09 ^{fg}	0.72 ± 0.01 ^{l}	2.38 ± 0.04 ^{f}	0.74 ± 0.03 ^{hi}	3.91 ± 0.04 ^{rg}	3.15 ± 0.04 ^{fghi}
		'Redbor F_1'	6.66 ± 0.21 ^{ef}	0.55 ± 0.01 ^{i}	2.08 ± 0.05 ^{de}	0.58 ± 0.03 ^{fg}	3.86 ± 0.02 ^{r}	2.99 ± 0.04 ^{efgh}
	8-OH-7-I-5QSA	'Oldenbor F_1'	6.56 ± 0.04 ^{ef}	0.71 ± 0.00 ^{l}	2.07 ± 0.05 ^{de}	0.59 ± 0.01 ^{g}	3.46 ± 0.05 ^{e}	2.66 ± 0.04 ^{cdef}
		'Redbor F_1'	6.23 ± 0.14 ^{e}	0.70 ± 0.01 ^{l}	1.93 ± 0.08 ^{cd}	0.43 ± 0.02 ^{d}	3.40 ± 0.09 ^{e}	2.65 ± 0.14 ^{cde}
	5-Cl-7-I-8-Q	'Oldenbor F_1'	5.32 ± 0.30 ^{d}	0.46 ± 0.02 ^{f}	1.70 ± 0.03 ^{c}	0.53 ± 0.03 ^{ef}	2.89 ± 0.01 ^{d}	2.38 ± 0.13 ^{cd}
		'Redbor F_1'	7.25 ± 0.13 ^{fg}	0.58 ± 0.01 ^{j}	2.30 ± 0.03 ^{ef}	0.56 ± 0.03 ^{efg}	4.15 ± 0.05 ^{g}	3.36 ± 0.13 ^{ghij}

Results are shown as mean ± standard error (SE); n = 3; homogeneous groups refer to a three-factor analysis of variance: factor No. 1 culinary treatment: raw, steaming, blanching, boiling x factor No. 2 type of enrichment: control, KIO_3, 8-OH-7-I-5QSA, 5-Cl-7-I-8-Q x factor No 3 kale cultivar: 'Oldenbor F_1' and 'Redbor F_1'.; means followed by the same letter are not significantly different $p < 0.05$ (Duncan's post-hoc test).

Figure S3. The antioxidant activity (**B**) and content of total polyphenols (**A**), total carotenoids (**D**), and ascorbic acid (**C**) in leaves curly kale 'Oldenbor F_1' and 'Redbor F_1' before and after heat treatment; means followed by different letters for treatments, differ significantly at $p < 0.05$ (Duncan's post-hoc test); bars indicate standard error (n = 3). Homogeneous groups refer to a three-factor analysis of variance: factor No. 1 culinary treatment: raw, steaming, blanching, boiling x factor No. 2 type of enrichment: control, KIO_3, 8-OH-7-I-5QSA, 5-Cl-7-I-8-Q x factor No. 3 kale cultivar: 'Oldenbor F_1' and 'Redbor F_1'.

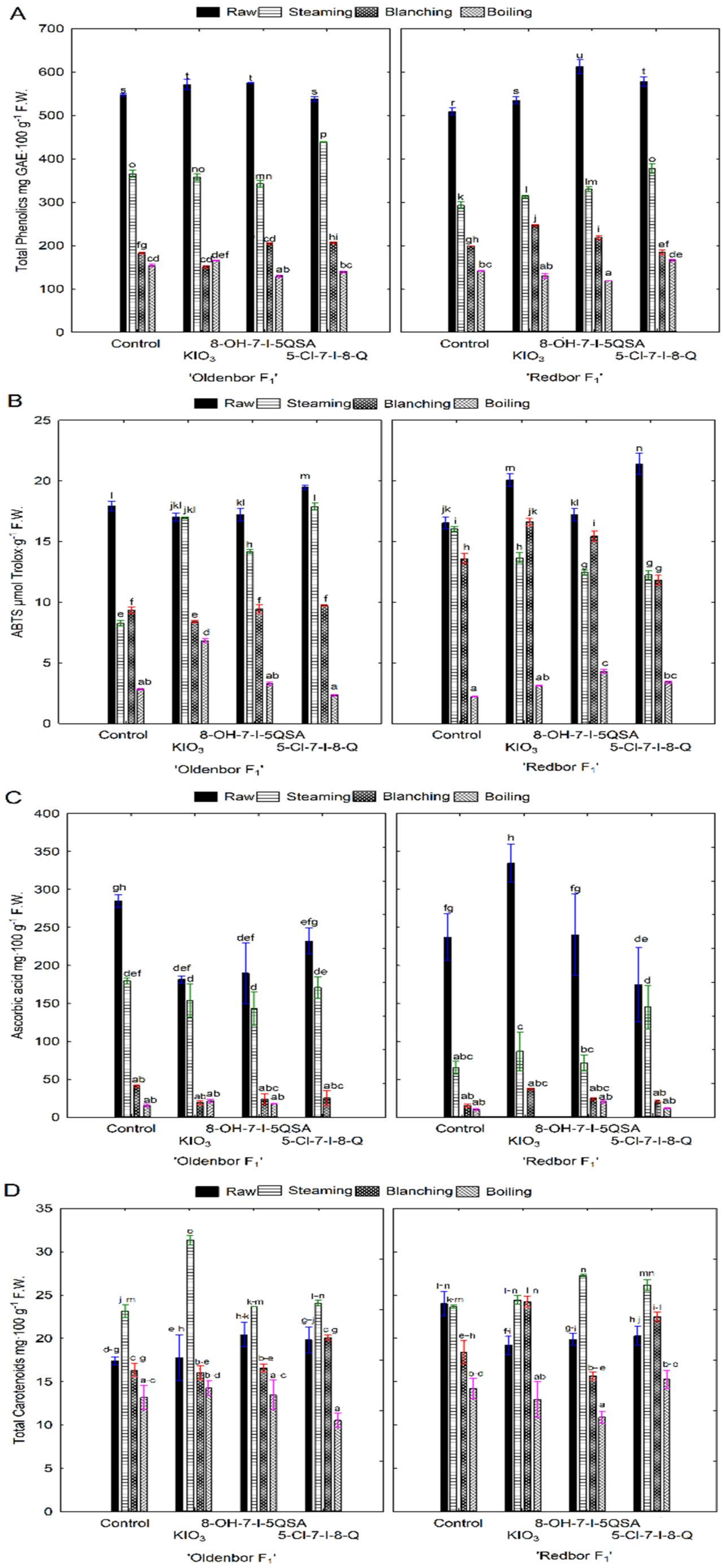

Table 4. The antioxidant activity and content of total phenolics, total carotenoids, and ascorbic acid in kale ‘Oldenbor F$_1$’ and ‘Redbor F$_1$‘ before and after heat treatments.

**Culinary treatment**	**Enrichment**	**Cultivar**	**Total Phenolics mg GAE·100 g^{-1} F.W.**	**ABTS$^{\bullet+}$ µmol Trolox·g^{-1} F.W.**	**Ascorbic Acid mg·100 g^{-1} F.W.**	**Total Carotenoids mg·100 g^{-1} F.W.**
Raw	Control	‘Oldenbor F$_1$’	547.71 ± 4.65 ^{s}	17.93 ± 0.67 ^{l}	284.97 ± 14.23 ^{gh}	17.46 ± 0.75 ^{defgh}
		‘Redbor F$_1$’	507.40 ± 13.72 ^{r}	16.57 ± 0.81 ^{jk}	237.01 ± 53.17 ^{fg}	24.00 ± 2.39 ^{lmn}
	KIO_3	‘Oldenbor F$_1$’	571.24 ± 19.79 ^{t}	17.03 ± 0.55 ^{jkl}	181.56 ± 7.94 ^{def}	17.83 ± 4.57 ^{efgh}
		‘Redbor F$_1$’	532.93 ± 14.16 ^{s}	20.10 ± 0.89 ^{m}	334.42 ± 43.57 ^{h}	19.19 ± 1.85 ^{fghi}
	8-OH-7-I-5QSA	‘Oldenbor F$_1$’	575.27 ± 2.33 ^{t}	17.23 ± 0.90 ^{kl}	189.73 ± 69.11 ^{def}	20.48 ± 2.47 ^{hijk}
		‘Redbor F$_1$’	610.89 ± 28.23 ^{u}	17.27 ± 0.87 ^{kl}	240.29 ± 92.65 ^{fg}	19.87 ± 1.20 ^{ghi}
	5-Cl-7-I-8-Q	‘Oldenbor F$_1$’	538.31 ± 8.79 ^{s}	19.47 ± 0.32 ^{m}	232.11 ± 30.12 ^{efg}	19.87 ± 2.57 ^{ghij}
		‘Redbor F$_1$’	575.94 ± 18.73 ^{t}	21.43 ± 1.53 ^{n}	174.61 ± 85.00 ^{de}	20.27 ± 1.88 ^{hij}
Steaming	Control	‘Oldenbor F$_1$’	365.38 ± 14.71 ^{o}	8.27 ± 0.40 ^{e}	179.89 ± 6.66 ^{def}	23.27 ± 1.27 ^{jklm}
		‘Redbor F$_1$’	292.15 ± 12.81 ^{k}	16.07 ± 0.35 ^{i}	65.67 ± 14.59 ^{abc}	23.66 ± 0.32 ^{klm}
	KIO_3	‘Oldenbor F$_1$’	358.60 ± 13.38 ^{no}	16.97 ± 0.15 ^{jkl}	153.89 ± 38.73 ^{d}	31.41 ± 0.89 ^{o}
		‘Redbor F$_1$’	312.47 ± 5.54 ^{l}	13.74 ± 0.72 ^{h}	86.62 ± 44.45 ^{c}	24.48 ± 0.87 ^{lmn}
	8-OH-7-I-5QSA	‘Oldenbor F$_1$’	342.47 ± 13.71 ^{mn}	14.17 ± 0.31 ^{h}	142.83 ± 37.91 ^{d}	23.75 ± 0.04 ^{klm}
		‘Redbor F$_1$’	328.82 ± 9.99 ^{lm}	12.47 ± 0.31 ^{g}	71.53 ± 17.75 ^{bc}	27.24 ± 0.21 ^{n}
	5-Cl-7-I-8-Q	‘Oldenbor F$_1$’	439.03 ± 1.48 ^{p}	17.87 ± 0.50 ^{l}	171.19 ± 24.41 ^{de}	24.15 ± 0.55 ^{lmn}
		‘Redbor F$_1$’	376.13 ± 19.28 ^{o}	12.31 ± 0.60 ^{g}	145.26 ± 50.03 ^{d}	26.16 ± 1.12 ^{mn}
Blanching	Control	‘Oldenbor F$_1$’	183.87 ± 2.96 ^{fg}	9.40 ± 0.46 ^{f}	41.46 ± 3.20 ^{ab}	16.52 ± 1.37 ^{cdefg}
		‘Redbor F$_1$’	199.03 ± 2.87 ^{gh}	13.68 ± 0.73 ^{h}	15.41 ± 3.35 ^{ab}	18.37 ± 2.35 ^{efgh}
	KIO_3	‘Oldenbor F$_1$’	152.26 ± 4.75 ^{cd}	8.38 ± 0.17 ^{e}	19.06 ± 4.89 ^{ab}	16.13 ± 1.41 ^{bcde}
		‘Redbor F$_1$’	244.62 ± 4.29 ^{j}	16.68 ± 0.51 ^{jk}	37.42 ± 1.70 ^{abc}	24.13 ± 1.09 ^{lmn}
	8-OH-7-I-5QSA	‘Oldenbor F$_1$’	204.95 ± 3.13 ^{hi}	9.48 ± 0.66 ^{f}	24.03 ± 12.24 ^{abc}	16.66 ± 0.79 ^{cdefg}
		‘Redbor F$_1$’	217.74 ± 6.83 ^{i}	15.52 ± 0.75 ^{i}	24.96 ± 2.30 ^{abc}	15.66 ± 0.81 ^{bcde}
	5-Cl-7-I-8-Q	‘Oldenbor F$_1$’	206.66 ± 3.36 ^{hi}	9.74 ± 0.12 ^{f}	25.40 ± 16.96 ^{abc}	20.16 ± 0.58 ^{hij}
		‘Redbor F$_1$’	179.14 ± 2.99 ^{ef}	11.92 ± 0.73 ^{g}	21.46 ± 2.06 ^{ab}	22.56 ± 0.92 ^{ijkl}
Boiling	Control	‘Oldenbor F$_1$’	154.30 ± 5.22 ^{cd}	2.82 ± 0.09 ^{ab}	15.71 ± 3.43 ^{ab}	13.23 ± 2.41 ^{abc}
		‘Redbor F$_1$’	140.32 ± 1.29 ^{bc}	2.26 ± 0.09 ^{a}	10.25 ± 2.18 ^{ab}	14.22 ± 2.04 ^{bcd}
	KIO_3	‘Oldenbor F$_1$’	166.02 ± 1.62 ^{def}	6.84 ± 0.25 ^{d}	21.31 ± 4.00 ^{ab}	14.31 ± 1.49 ^{bcd}
		‘Redbor F$_1$’	125.48 ± 4.03 ^{ab}	3.16 ± 0.09 ^{ab}	0.00 ± 0.00 ^{a}	12.96 ± 3.61 ^{ab}
	8-OH-7-I-5QSA	‘Oldenbor F$_1$’	129.36 ± 5.33 ^{ab}	3.30 ± 0.18 ^{ab}	17.79 ± 0.20 ^{ab}	13.52 ± 3.01 ^{abc}
		‘Redbor F$_1$’	118.39 ± 0.65 ^{a}	4.34 ± 0.27 ^{c}	20.73 ± 3.73 ^{ab}	10.89 ± 1.20 ^{a}
	5-Cl-7-I-8-Q	‘Oldenbor F$_1$’	139.35 ± 2.59 ^{bc}	2.34 ± 0.09 ^{a}	0.00 ± 0.00 ^{a}	10.59 ± 1.42 ^{a}
		‘Redbor F$_1$’	164.41 ± 4.40 ^{de}	3.44 ± 0.18 ^{bc}	11.81 ± 1.25 ^{ab}	15.27 ± 1.83 ^{bcde}

Results are shown as mean ± standard error (SE); n = 3; homogeneous groups refer to a three-factor analysis of variance: factor No. 1 culinary treatment: raw, steaming, blanching, boiling x factor No. 2 type of enrichment: control, KIO_3, 8-OH-7-I-5QSA, 5-Cl-7-I-8-Q x factor No. 3 kale cultivar 'Oldenbor F$_1$' and 'Redbor F$_1$'; means followed by the same letter are not significantly different $p < 0.05$ (Duncan’s post-hoc test).

Figure S4. Percentage coverage of Recommended Daily Allowance for iodine (% RDA-I) and hazard quotient (HQ) for intake of iodine through consumption of 100 g and 50 g portions of leaves curly kale 'Oldenbor F_1' and 'Redbor F_1' before and after heat treatment in individual by adults 70 kg body weight; means followed by different letters for treatments, differ significantly at $p < 0.05$ (Duncan's post-hoc test); bars indicate standard error (n = 4). **A.** Daily Intake of I with 50 g of kale (μg I·day^{-1}); **B.** Daily Intake of I with 100 g of kale (μg I·day^{-1}); **C.** % RDA I (in 50 g portion of kale); **D.** % RDA I (in 100 g portion of kale); **E.** HQ for 50 g portion of kale; **F.** HQ for 100 g portion of kale. Homogeneous groups refer to a three-factor analysis of variance: factor No. 1 culinary treatment: raw, steaming, blanching, boiling x factor No. 2 type of enrichment: control, KIO_3, 8-OH-7-I-5QSA, 5-Cl-7-I-8-Q x factor No. 3 kale cultivar: 'Oldenbor F_1' and 'Redbor F_1'.

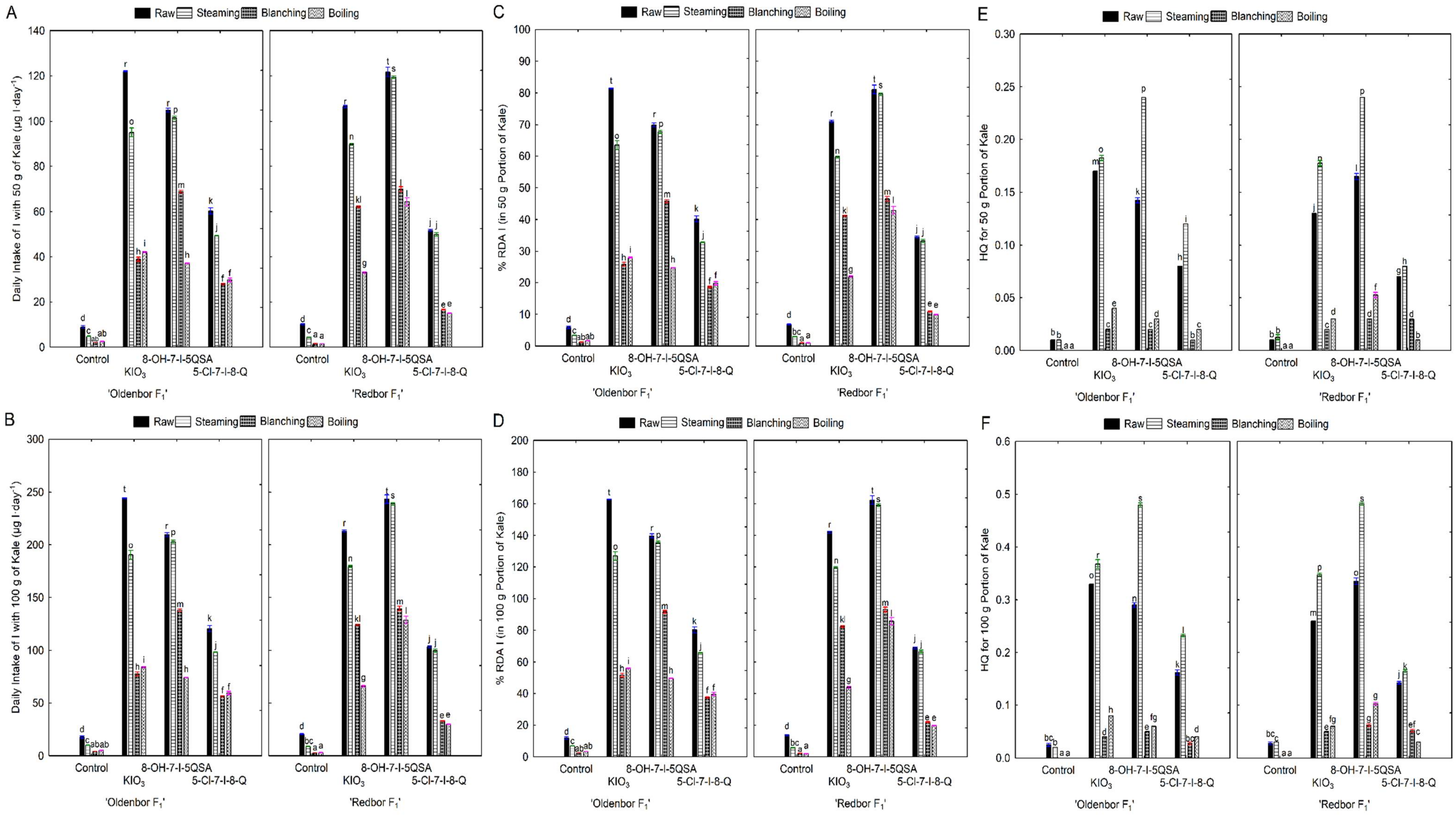

Table 5. Percentage coverage of Recommended Daily Allowance for iodine (% RDA-I) and hazard quotient (HQ) for intake of iodine through consumption of 100 g and 50 g portions of kale 'Oldenbor F$_1$' and 'Redbor F$_1$' before and after heat treatment in individual by adults 70 kg body weight.

Culinary treatment	Enrichment	Cultivar	Daily Intake of I with 50 g of kale (µg I·day^{-1})	Daily Intake of I with 100 g of kale (µg I·day^{-1})	% RDA I (in 50 g portion of kale)	% RDA I (in 100 g portion of kale)	HQ for 50 g portion of kale	HQ for 100 g portion of kale
Raw	Control	'Oldenbor F$_1$'	8.93 ± 1.18 ^{d}	17.85 ± 2.37 ^{d}	5.95 ± 0.79 ^{d}	11.90 ± 1.58 ^{d}	0.012 ± 0.00 ^{b}	0.024 ± 0.01 ^{bc}
		'Redbor F$_1$'	10.23 ± 0.54 ^{d}	20.45 ± 1.09 ^{d}	6.82 ± 0.36 ^{d}	13.63 ± 0.72 ^{d}	0.013 ± 0.00 ^{b}	0.026 ± 0.01 ^{bc}
	KIO_3	'Oldenbor F$_1$'	122.13 ± 0.36 ^{t}	244.26 ± 0.72 ^{t}	81.42 ± 0.24 ^{t}	162.84 ± 0.48 ^{t}	0.167± 0.00 ^{m}	0.333 ± 0.00 ^{o}
		'Redbor F$_1$'	106.55 ± 1.04 ^{r}	213.10 ± 2.07 ^{r}	71.03 ± 0.69 ^{r}	142.07 ± 1.38 ^{r}	0.131 ± 0.00 ^{j}	0.263 ± 0.00 ^{m}
	8-OH-7-I-5QSA	'Oldenbor F$_1$'	104.90 ± 1.88 ^{r}	209.80 ± 3.77 ^{r}	69.93 ± 1.26 ^{r}	139.87 ± 2.51 ^{r}	0.144 ± 0.01 ^{k}	0.289 ± 0.01 ^{n}
		'Redbor F$_1$'	121.74 ± 4.11 ^{t}	243.47 ± 8.21 ^{t}	81.16 ± 2.74 ^{t}	162.31 ± 5.48 ^{t}	0.168 ± 0.01 ^{l}	0.335 ± 0.01 ^{o}
	5-Cl-7-I-8-Q	'Oldenbor F$_1$'	60.30 ± 2.87 ^{k}	120.60 ± 5.75 ^{k}	40.20 ± 1.92 ^{k}	80.40 ± 3.83 ^{k}	0.081 ± 0.00 ^{h}	0.161 ± 0.01 ^{k}
		'Redbor F$_1$'	51.70 ± 0.91 ^{j}	103.40 ± 1.82 ^{j}	34.47 ± 0.60 ^{j}	68.93 ± 1.21 ^{j}	0.071 ± 0.00 ^{g}	0.142 ± 0.01 ^{j}
Steaming	Control	'Oldenbor F$_1$'	5.16 ± 0.17 ^{c}	10.33 ± 0.33 ^{c}	3.44 ± 0.11 ^{c}	6.88 ± 0.22 ^{c}	0.012 ± 0.00 ^{b}	0.024 ± 0.00 ^{b}
		'Redbor F$_1$'	4.46 ± 0.09 ^{c}	8.91 ± 0.17 ^{bc}	2.97 ± 0.06 ^{bc}	5.94 ± 0.12 ^{bc}	0.015 ± 0.01 ^{b}	0.029 ± 0.00 ^{c}
	KIO_3	'Oldenbor F$_1$'	95.20 ± 4.13 ^{o}	190.40 ± 8.25 ^{o}	63.47 ± 2.75 ^{o}	126.93 ± 5.50 ^{o}	0.182 ± 0.01 ^{o}	0.365 ± 0.02 ^{r}
		'Redbor F$_1$'	89.69 ± 0.91 ^{n}	179.39 ± 1.82 ^{n}	59.80 ± 0.61 ^{n}	119.59 ± 1.21 ^{n}	0.175 ± 0.00 ^{n}	0.350 ± 0.01 ^{p}
	8-OH-7-I-5QSA	'Oldenbor F$_1$'	101.58 ± 1.48 ^{p}	203.16 ± 2.96 ^{p}	67.72 ± 0.99 ^{p}	135.44 ± 1.97 ^{p}	0.240 ± 0.00 ^{p}	0.481 ± 0.01 ^{s}
		'Redbor F$_1$'	119.40 ± 1.05 ^{s}	238.80 ± 2.09 ^{s}	79.60 ± 0.70 ^{s}	159.20 ± 1.39 ^{s}	0.240 ± 0.00 ^{p}	0.481 ± 0.01 ^{s}
	5-Cl-7-I-8-Q	'Oldenbor F$_1$'	49.37 ± 0.33 ^{j}	98.73 ± 0.66 ^{j}	32.91 ± 0.22 ^{j}	65.82 ± 0.44 ^{j}	0.117 ± 0.00 ^{i}	0.233 ± 0.00 ^{l}
		'Redbor F$_1$'	49.92 ± 1.58 ^{j}	99.84 ± 3.16 ^{j}	33.28 ± 1.05 ^{j}	66.56 ± 2.11 ^{j}	0.081 ± 0.00 ^{h}	0.162 ± 0.01 ^{k}
Blanching	Control	'Oldenbor F$_1$'	2.17 ± 0.05 ^{ab}	4.34 ± 0.10 ^{ab}	1.45 ± 0.04 ^{ab}	2.90 ± 0.07 ^{ab}	0.001 ± 0.00 ^{a}	0.002 ± 0.00 ^{a}
		'Redbor F$_1$'	1.67 ± 0.07 ^{a}	3.34 ± 0.13 ^{a}	1.12 ± 0.05 ^{a}	2.23 ± 0.09 ^{a}	0.002 ± 0.00 ^{a}	0.003 ± 0.00 ^{a}
	KIO_3	'Oldenbor F$_1$'	38.97 ± 1.79 ^{h}	77.94 ± 3.59 ^{h}	25.98 ± 1.20 ^{h}	51.96 ± 2.39 ^{h}	0.018 ± 0.00 ^{c}	0.037 ± 0.00 ^{d}
		'Redbor F$_1$'	62.15 ± 0.78 ^{kl}	124.30 ± 1.56 ^{kl}	41.43 ± 0.52 ^{kl}	82.86 ± 1.04 ^{kl}	0.025 ± 0.00 ^{c}	0.049 ± 0.00 ^{e}
	8-OH-7-I-5QSA	'Oldenbor F$_1$'	68.87 ± 1.31 ^{m}	137.74 ± 2.63 ^{m}	45.91 ± 0.88 ^{m}	91.82 ± 1.75 ^{m}	0.023 ± 0.00 ^{c}	0.046 ± 0.00 ^{e}
		'Redbor F$_1$'	69.89 ± 2.27 ^{m}	139.78 ± 4.53 ^{m}	46.60 ± 1.51 ^{m}	93.19 ± 3.03 ^{m}	0.032 ± 0.00 ^{d}	0.063 ± 0.01 ^{g}
	5-Cl-7-I-8-Q	'Oldenbor F$_1$'	28.11 ± 0.66 ^{f}	56.22 ± 1.32 ^{f}	18.74 ± 0.44 ^{f}	37.48 ± 0.88 ^{f}	0.013 ± 0.00 ^{b}	0.026 ± 0.01 ^{bc}
		'Redbor F$_1$'	16.58 ± 0.41 ^{e}	33.16 ± 0.82 ^{e}	11.06 ± 0.28 ^{e}	22.11 ± 0.54 ^{e}	0.027 ± 0.00 ^{d}	0.054 ± 0.01 ^{ef}
Boiling	Control	'Oldenbor F$_1$'	2.60 ± 0.04 ^{ab}	5.19 ± 0.08 ^{ab}	1.73 ± 0.03 ^{ab}	3.46 ± 0.05 ^{ab}	0.002 ± 0.00 ^{a}	0.005 ± 0.00 ^{a}
		'Redbor F$_1$'	1.54 ± 0.05 ^{a}	3.08 ± 0.10 ^{a}	1.03 ± 0.03 ^{a}	2.05 ± 0.07 ^{a}	0.001 ± 0.00 ^{a}	0.003 ± 0.00 ^{a}
	KIO_3	'Oldenbor F$_1$'	42.11 ± 0.43 ^{i}	84.21 ± 0.86 ^{i}	28.07 ± 0.28 ^{i}	56.14 ± 0.57 ^{i}	0.040 ± 0.00 ^{e}	0.080 ± 0.00 ^{h}
		'Redbor F$_1$'	32.94 ± 0.80 ^{g}	65.88 ± 1.60 ^{g}	21.96 ± 0.54 ^{g}	43.92 ± 1.07 ^{g}	0.028 ± 0.00 ^{d}	0.057 ± 0.00 ^{fg}
	8-OH-7-I-5QSA	'Oldenbor F$_1$'	37.30 ± 0.18 ^{h}	74.60 ± 0.36 ^{h}	24.87 ± 0.12 ^{h}	49.73 ± 0.24 ^{h}	0.032 ± 0.00 ^{d}	0.064 ± 0.00 ^{fg}
		'Redbor F$_1$'	64.39 ± 3.53 ^{l}	128.78 ± 7.05 ^{l}	42.93 ± 2.35 ^{l}	85.85 ± 4.70 ^{l}	0.052 ± 0.01 ^{f}	0.104 ± 0.01 ^{g}
	5-Cl-7-I-8-Q	'Oldenbor F$_1$'	29.69 ± 1.55 ^{f}	59.38 ± 3.10 ^{f}	19.79 ± 1.03 ^{f}	39.58 ± 2.06 ^{f}	0.021 ± 0.00 ^{c}	0.041 ± 0.00 ^{d}
		'Redbor F$_1$'	14.97 ± 0.21 ^{e}	29.93 ± 0.43 ^{e}	9.98 ± 0.14 ^{e}	19.96 ± 0.28 ^{e}	0.014 ± 0.00 ^{b}	0.028 ± 0.00 ^{c}

Results are shown as mean ± standard error (SE); n = 4; homogeneous groups refer to a three-factor analysis of variance factor No. 1 culinary treatment: raw, steaming, blanching, boiling x factor No. 2 type of enrichment: control, KIO_3, 8-OH-7-I-5QSA, 5-Cl-7-I-8-Q x factor No. 3 kale cultivar: 'Oldenbor F$_1$' and 'Redbor F$_1$'; means followed by the same letter are not significantly different $p < 0.05$ (Duncan's post-hoc test).
